# *Erwinia amylovora* Novel Plasmid pEI70: Complete Sequence, Biogeography, and Role in Aggressiveness in the Fire Blight Phytopathogen

**DOI:** 10.1371/journal.pone.0028651

**Published:** 2011-12-09

**Authors:** Pablo Llop, Jordi Cabrefiga, Theo H. M. Smits, Tanja Dreo, Silvia Barbé, Joanna Pulawska, Alain Bultreys, Jochen Blom, Brion Duffy, Emilio Montesinos, María M. López

**Affiliations:** 1 Instituto Valenciano de Investigaciones Agrarias, IVIA, Moncada, Valencia, Spain; 2 Institute of Food and Agricultural Technology, INTEA, CIDSAV-CeRTA, University of Girona, Girona, Spain; 3 Agroscope Changins-Wädenswil, ACW, Swiss National Competence Center for Fire Blight, Wädenswil, Switzerland; 4 National Institute of Biology, NIB, Večna pot 111, SI-1000 Ljubljana, Slovenia; 5 Institute of Horticulture, Skierniewice, Poland; 6 Département Sciences du Vivant, Centre Wallon de Recherches Agronomiques, Gembloux, Belgium; 7 CeBiTec, Bielefeld University, Bielefeld, Germany; University of Wisconsin-Milwaukee, United States of America

## Abstract

Comparative genomics of several strains of *Erwinia amylovora*, a plant pathogenic bacterium causal agent of fire blight disease, revealed that its diversity is primarily attributable to the flexible genome comprised of plasmids. We recently identified and sequenced in full a novel 65.8 kb plasmid, called pEI70. Annotation revealed a lack of known virulence-related genes, but found evidence for a unique integrative conjugative element related to that of other plant and human pathogens. Comparative analyses using BLASTN showed that pEI70 is almost entirely included in plasmid pEB102 from *E. billingiae*, an epiphytic *Erwinia* of pome fruits, with sequence identities superior to 98%. A duplex PCR assay was developed to survey the prevalence of plasmid pEI70 and also that of pEA29, which had previously been described in several *E. amylovora* strains. Plasmid pEI70 was found widely dispersed across Europe with frequencies of 5–92%, but it was absent in *E. amylovora* analyzed populations from outside of Europe. Restriction analysis and hybridization demonstrated that this plasmid was identical in at least 13 strains. Curing *E. amylovora* strains of pEI70 reduced their aggressiveness on pear, and introducing pEI70 into low-aggressiveness strains lacking this plasmid increased symptoms development in this host. Discovery of this novel plasmid offers new insights into the biogeography, evolution and virulence determinants in *E. amylovora*.

## Introduction

*Erwinia amylovora* is the causal agent of fire blight, the most serious disease that affects pome fruit trees worldwide [1]. This bacterium has been studied for several decades, and it has been found that *E. amylovora* can infect a wide range of hosts within the family *Rosaceae* (apple, pear, quince, loquat, and many ornamentals) [2] compared to other *Erwinia* species pathogenic to pome fruit trees (e.g., *E. pyrifoliae*, *E. piriflorinigrans*) [3], [4]. Although phenotypic traits can be different in *E. amylovora* isolates (e.g., metabolic activity, virulence) [5]–[8], several genetic analyses have demonstrated that it is a very homogeneous species [9]–[12]. The genes involved in pathogenicity, virulence and related behavior are highly conserved and rarely show significant differences in sequence or structure. Whole-genome sequence analysis has demonstrated that the two available genome sequences have over 99.99% nucleotide sequence identity [13], which is in accordance with the high homogeneity that this species presents. This is supported by the preliminary analysis of four further genome sequences from European isolates that showed that, apart from plasmid content, the pan-genome of *E. amylovora* is closed [14].

The most obvious difference between the *E. amylovora* genomes of the sequenced strains CFBP 1430 and Ea 273 is the presence of plasmid pEA72 in the latter strain [13], [15]. Plasmids also appear to be the main elements contributing to diversity within the pan-genome of *E. amylovora* [14]. However, although a total of 14 plasmids have been detected in isolates of this species [16]–[20] (Table 1), in practice, knowledge of the existence of this extra-chromosomal material is limited to some strains and few plasmids. A reason for the lack of information on the presence of plasmids could be that no intensive screening analyses have been performed worldwide to determine the plasmid content in *E. amylovora* strain collections from a large number of countries. Apart from the near-ubiquitous plasmid pEA29, whose role in *E. amylovora* is still unclear but includes biosynthesis of thiamine [21], plasmids of 8.7 kb and 34 kb size have been assigned to confer resistance to streptomycin, and a plasmid of 2.8 kb contains a gene with high sequence identity to a β-lactamase that could confer resistance to ampicillin [16], [22], [23]. All other plasmids reported in Table 1 are cryptic.

**Table 1:** Other plasmids different from pEI70 that have been found and studied in different *E. amylovora* strains.

Name	Strain(s)	Host	Origin	Features**	Accession	References
					Number	
pEA72	Ea273	Apple	NY, USA	Cryptic	FN666577	[15]
pEL60	LebB66	Apple, pear	Lebanon	Cryptic	NC_005246	[20]
pEA34	CA11	Apple/pear	Michigan, USA	Str^R^	-	[16]
pEU30	UTRJ2	Apple/pear	Utha, USA	Cryptic	NC_005247	[20]
pEA29*	Ea88	Pear	Washington, USA	Thiamine	AF264948	[40]
pEA29*	Ea273	Apple	NY, USA	Thiamine	FN666576	[15]
pEA29*	CFBP 1430	*Crataegus* sp.	France	Thiamine	FN43411	[13]
pEA29*	ATCC BAA-2158	*Rubus* sp.	Illinois, USA	Thiamine	FR719212	[50]
pEA8.7	CA3R	Apple	California, USA	Str^R^	-	[22]
pEAR5.2	ATCC BAA-2158	*Rubus* sp.	Illinois, USA	Cryptic	FR719211	[50]
pEAR4.3	ATCC BAA-2158	*Rubus* sp.	Illinois, USA	Cryptic	FR719210	[50]
pEA2.8	IL-5	*Rubus* sp.	Illinois, USA	Amp^R^	AY123047	[23]
pEA1.7	IH3-1	*Crataegus* sp.	Louisiana, USA	Cryptic	AY123046	[23]

Three plasmids (one from strain IH3-1 and two other from *Rubus* strain IL5) have been reported, but names and sequence information are unavailable and they are not included.

*: plasmid pEA29 is present in almost all *E. amylovora* strains. Here, only the four plasmids that have been sequenced and their hosts are shown.

**: Str^R^: resistance to streptomycin; Thiamine: thiamine biosynthesis; Amp^R^: possible resistance to ampicillin. Not functionally demonstrated. Cryptic: plasmids that have no known function, with no apparent effect on the phenotype of its host cell.

Despite studies having elucidated the effect of plasmid pEA29 [17], [18], [21], the presence/absence of this plasmid alone does not explain the differences in aggressiveness observed among *E. amylovora* isolates. Recently, a strain obtained from a fire blight outbreak in Spain was shown to lack pEA29 but it harbors a different plasmid of approximately 70 kb [24]. This plasmid, designated pEI70, was sequenced and annotated in the present study. We analyzed its presence in a wide collection of isolates from different geographic origins, hosts and years. It was shown that pEI70 was present at different percentages in isolates from European countries, but not in isolates from elsewhere. Furthermore, its influence on aggressiveness was examined.

## Materials and Methods

### Bacterial strains, plasmids and growth media

The *E. amylovora* isolates analyzed for plasmid content, the vectors employed for cloning pEI70 and the bacteria used for virulence analyses and genetic comparisons are described in Table 2. Bacteria were cultured on LB agar, supplemented with antibiotics as appropriate for mutants, and incubated at 26°C (*E. amylovora*) or 37°C (*Escherichia coli*). Throughout, the following antibiotic concentrations were used for: *E. coli*, kanamycin 50 µg/ml; and for *E. amylovora*, streptomycin 25 µg/ml and kanamycin 50 µg/ml. When required, nicotinic acid (1 mg/ml) and thiamine (0.2%) were added to M9 minimal broth medium (mmT) [25].

**Table 2:** *Erwinia amylovora* strains analyzed for plasmid content and for genetic comparison, and plasmids employed in the different experiments.

Bacterial strains	Host	Plasmid content	Origin/year	References
			of isolation	
CFBP 1430	*Crataegus* sp.	pEA29	France/1972	[51]
PMV 6014	-	-	France	[17]
Ea 273	Apple	pEA29, pEA72	USA/1971	[52]
IVIA 1614-1	*Pyracantha* sp.	pEA29, pEI70	Spain/1996	This work
IVIA 1614-2a	*Crataegus* sp.	pEI70	Spain/1996	[24]
IVIA 1596	Pear	pEI70	Spain/1996	This work
BC3	Apple	-	Serbia/2003	[46]
CGJ2	Apple	-	Serbia/2003	[46]
E 70	*Cotoneaster* sp.	pEA29, pEI70, 30 kb plasmid	Ireland/1997	[53]
NCPPB 3299	*Pernettya* sp.	pEI70	UK/1983	[47]
IVIA 1614-2a-pEI70	-	-	Spain/2005	This work
IVIA 1596-pEI70	-	-	Spain/2005	This work
ACW 56400	Pear	PEA29, pEI70	Switzerland/	[31]
			2007	

Plasmids	Characteristics		Origin	References
pBBRMCS2	Kan^R^, cloning vector	-	USA	[54]
pEA29::Tn5393	Str^R^	-	USA	[26]
pEI70::Tn5	Kan^R^, labelled	-	Spain	This work

### Characteristics of plasmid pEI70

Stability analyses were performed following the protocol described in Foster et al., [20], consisting of a 200-generation serial transfer experiment in mmT medium. Briefly, 10 ml of a bacterial suspension were grown up for 24 h at 26°C, and 10 µl were transferred to a new 10 ml tube of the same medium and incubated again. These transfers were performed over 20 consecutive days. We employed one strain harboring only pEI70 (IVIA 1614-2a), and another strain that carries pEA29 in addition to pEI70 (IVIA 1614-1), to observe the possible influence of pEA29 on the stability of pEI70. One hundred colonies were taken after 20 days and checked for the presence of pEI70. Additionally, 25 colonies were analyzed for plasmid content by plasmid extraction and by restriction analysis to confirm that the plasmid had not integrated into the chromosome.

Plasmid pEI70 was marked with a transposon with resistance to kanamycin using the EZ-Tn5 Insertion kit (Epicentre, Madison, WI, USA) following the manufacturer's instructions, yielding plasmid pEI70::Tn5. Conjugative analysis was performed using two derivatives of plasmid free *E amylovora* strain CGJ2. One carried the plasmid pEA29::Tn5393 from strain MI11-1, a wild-type strain harboring pEA29 with a natural transposon which confers resistance to streptomycin, isolated from apple [26]. The other CGJ2 derivative contained plasmid pEI70::Tn5. Suspensions were adjusted to 10^7^ cfu/ml of each strain and 250 µl of each suspension were placed together on a 0.22 µm filter placed on a LB plate containing both antibiotics. On similar filters, one suspension of each strain was plated separately as controls. After 48 h incubation at 26°C, colonies appeared only in the mixed suspensions, and were analyzed for plasmid content by PCR using the duplex system.

### Sequence analysis of pEI70

Plasmid pEI70 from Spanish strain IVIA 1614-2a was sequenced using shotgun cloning and Sanger sequencing. The coverage was 13.0× with 929 reads (average read length 922 bp) in a single contig. Genes on pEI70 were predicted using a combined strategy [27] based on the CDS prediction programs Glimmer [28] and Critica [29]. Subsequently, the potential function of each predicted gene was automatically assigned using the GenDB annotation pipeline [30]. The resulting plasmid annotation was manually curated. Routine sequence manipulations were completed using several subroutines of the LASERGENE package (DNASTAR, Madison, WI, USA). In the framework of a larger genome sequencing project (Powney *et al*, unpublished), the genome of a recent Swiss isolate *E. amylovora* strain ACW 56400 [31], was sequenced using Illumina sequencing. This genome contained an identical copy of pEI70 next to plasmid pEA29. The sequence of pEI70 from *E. amylovora* strain ACW 56400, identical in nucleotide sequence to strain IVIA 1614-2a, is available at NCBI under accession number CP002951.

### Analyses of plasmid content by PCR and extraction analyses

*E. amylovora* strains were analyzed for the presence of pEA29 by PCR using primers AJ75 and AJ76 of McManus and Jones [32]. To screen plasmid pEI70, a conventional PCR protocol was designed, using primers 1.7F (5′-CCCCGTGAACAACAGACCACC-3′) and 1.7R (5′-AATCTGACAGCCGCAACCCG-3′) derived from the sequence of a 1.7 kb *Bam*HI fragment of plasmid pEI70. Briefly, PCR reaction mix was as follows: 1× buffer (Tris-HCl 75 mM; KCl 50 mM; (NH_4_)_2_SO_4_ 20 mM); 0.1 mM dNTPs; 1.5 mM MgCl_2_; 5 µM primer 1.7F; 5 µM primer 1.7R; 1 U DNA polymerase (Biotools, Madrid, Spain). The PCR amplification conditions were 3 min at 94°C initial denaturation followed by 40 cycles of 45 s at 94°C, 40 s at 60°C and 1 min at 72°C, with a final extension at 72°C for 10 min.

Additionally, a duplex PCR using both pairs of primers was developed to allow detection of pEA29 and pEI70 in a single assay. The reaction mix for the duplex PCR was: 1× buffer; 0.1 mM dNTPs; 1.5 mM MgCl_2_; 5 µM primers AJ75-AJ76; 2.5 µM primers 1.7F-1.7R; 1 U DNA polymerase. The PCR conditions were identical to those of the single PCR. PCR products were 800 bp for pEA29 and 539 bp for pEI70 amplicons, analyzed on 1.2% agarose gels. Isolates providing deviant results by PCR (strains negative for pEA29 plasmid, positive for pEI70, or negative for both plasmids) were subjected to plasmid extraction using the protocol of Zhou *et al.* [33] to verify the lack and/or presence of the corresponding plasmids. With 13 isolates positive for pEI70 from different origins chosen at random, restriction analysis was performed using the enzyme *Bam*HI which generates only one band for pEA29 and 14 bands for pEI70, to confirm that the plasmid was identical in all isolates. Plasmid restrictions were analyzed on 0.8% agarose gels.

### Presence of plasmid pEI70 in *E. amylovora* strains from geographically different locations

Using the duplex PCR system, 1,480 *E. amylovora* strains from different collections from 21 countries in Europe, North America and Asia were analyzed to examine the distribution of strains that harbor pEI70 and whether strains without pEA29 are widespread in nature. Several isolates were obtained from international and laboratory collections and others were analyzed in laboratories in Belgium, Poland, Slovenia, Spain and Switzerland (Table 3).

**Table 3:** Screening of presence of plasmids pEA29 and pEI70 in strains of different international and collections from Institutes and laboratories using the duplex PCR system.

European	Strains	Strains with	% of strains	Strains without	Strains without
Countries	analyzed	pEI70	with pEI70	pEA29	any plasmid
Austria	25	0	-	0	0
Belgium	70	65	92.8	2	1
Bulgaria	4	0	-	0	0
Czech Republic	2	1	50.0	0	0
France	43	3	6.9	0	0
Germany	12	0	-	0	0
Greece	15	0	-	0	0
Hungary	10	0	-	0	0
Ireland	14	12	85.7	0	0
Italy	5	3	60.0	0	0
The Netherlands	4	2	50.0	0	0
Poland	120	7	5.8	0	0
Serbia	2	0	-	2	2
Slovenia	526	331	62.9	0	0
Spain	142	20	14.0	4	3
Switzerland	247	13	5.2	0	0
UK	8	2	25.0	1	0
Total	1249	458	34.6	9 (0.7%)	6 (0.48%)

Non European	Strains	Strains with	% of strains	Strains without	Strains without
countries	analyzed	pEI70	with pEI70	pEA29	plasmids
Canada	36	0	-	0	0
Lebanon	6	0	-	0	0
USA	117	0	-	0	0
Turkey	72	0	-	0	0
Total	231	0	-	0	0

Additional information is provided in Tables S2 and S3.

### Transformation of *E. amylovora* strains

Plasmids pEI70::Tn5 and pEA29::Tn5393 were introduced into *E. amylovora* strains by electroporation. Plasmids were introduced into the low aggressive strains BC3 and CGJ2 (Table 2), and also into strain PMV 6014 (strain CFBP 1430 cured of plasmid pEA29) that was employed as a control in the inoculation experiments (Table 4). Strains CGJ2+pEI70::Tn5 and CGJ2+pEA29::Tn5393 were also employed for the conjugation assays.

**Table 4:** Strains analyzed for aggressiveness on basis of severity of infections (0–100%) produced in immature pear fruits before and after introducing tagged plasmids pEA29::Tn5393 and/or pEI70::Tn5.

Strains analyzed	Aggressiveness	Plasmid	Plasmids	Aggressiveness after
		content	introduced	plasmid introduction
CFBP 1430*	92.1	pEA29	-	-
PMV 6014	47.5	-	pEI70	85.3
IVIA 1614-2a	70.6	pEI70	pEA29	79.8
IVIA 1596	83.1	pEI70	pEA29	82.8
BC3	22.2	-	pEA29	54.7
BC3	13.2	-	pEI70	75.0
CGJ2	20.0	-	pEA29	57.2
CGJ2	15.6	-	pEI70	68.6

*: positive control.

### Construction of plasmid-less strains

Plasmid pEI70 was eliminated from strains IVIA 1614-2a and IVIA 1596 using a subclone containing a 1 kb *Asu*I-*Eco*RI fragment that includes the *repA* gene in vector pBBR-MCS2. After introduction of the vector into the strains, the resulting kanamycin-resistant transformants were subcultured several times on LB with kanamycin. Individual colonies were screened for the loss of pEI70 by plasmid extraction and visualization on a 0.8% agarose gel. The cured strains were analyzed for aggressiveness compared to the same strains harboring the plasmid.

### Analysis of aggressiveness using *ex vivo* plant assays

Several analyses were performed to evaluate the aggressiveness of the different types of strains under study and to observe how the introduction of plasmids pEI70 and pEA29 affected this feature. The consequence of curing pEI70 from some strains harboring only this plasmid was also assessed. The material used in the different assays consisted of: i) strains that do not harbor any plasmid as confirmed by PCR and plasmid extraction, ii) the above strains with plasmid pEA29 and/or pEI70 introduced by electroporation, and iii) wild strains with only pEI70 (Table 4).

The first assay was performed to test the effect of plasmid pEA29 (pEA29::Tn5393), [26] when introduced into strains that do not harbor this plasmid and also in strains harboring only plasmid pEI70, and, further, to test the effect of curing pEI70 from strains harboring it alone (Table 4). The assay was performed using ‘Passe Crassane’ immature pear fruits. Briefly, pear fruits of 2–3 cm diameter, collected at 6-weeks after fruit set were washed in running water and soaked in a 1% sodium hypochlorite solution for 20 min. Subsequently, they were washed with sterile water and left to remove excessive water on a sterile bench. Bacterial suspensions were adjusted to 0.1 OD_600_ (10^8^ cfu/ml) and diluted to the corresponding concentration. Two concentrations were tested, 10^5^ cfu/ml and 10^6^ cfu/ml. Four wounds were performed with a pipette tip on each pear, and 10 µl of the diluted suspension placed on each wound. Then, fruits were placed in polystyrene trays, covered with plastic bags to maintain conditions of high humidity, and incubated at 25°C. The experimental design consisted of three repetitions of three pears (nine fruits) per strain and dose.

The incidence and severity of infected wounds (%) for each repetition (3 pears×4 wounds each) was assessed after 5 days of incubation. Wounds were considered as infected when either drops of bacterial exudates or necrosis appeared in and around the inoculation site. Severity of symptoms was evaluated by means of a visual scale (from 0 to 3). The scale was based on necrosis progression as follows: 0 = no symptoms, 1 = exudates located at the inoculation point, 2 = necrosis affecting area around the wound, 3 = necrosis expanding through the fruit. Disease severity (S) was calculated according to the following formula:
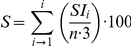
where *S* is the disease severity, *SI* is the corresponding severity index in an inoculated wound, *i* is the wound number, *n* the total wounds inoculated and *3* the maximum severity index.

A second assay was also performed in immature ‘Passe Crassane’ pear fruits to test the effect of the introduction of plasmid pEI70::Tn5 in strains lacking pEA29. The inoculation methodology and the experimental design were the same as described above for the first assay. The assay was performed at 10^5^ cfu/ml and 5×10^5^ cfu/ml pathogen concentrations. The incidence and severity of infected wounds (%) for each repetition (3 pears×4 wounds each) was assessed after 7 days incubation.

### Statistical analysis

ANOVA was performed to analyze the effect of each strain treatment on infection incidence and means were separated by the Tukey's test at *P*≤0.05. The analysis was done with the GLM procedure of the PC-Statistical Analysis System version 8.2 (SAS Institute Inc., Cary, NC, USA).

## Results

### Identification and sequencing of pEI70 in *E. amylovora* strain IVIA 1614-2a

**Figure 1:**
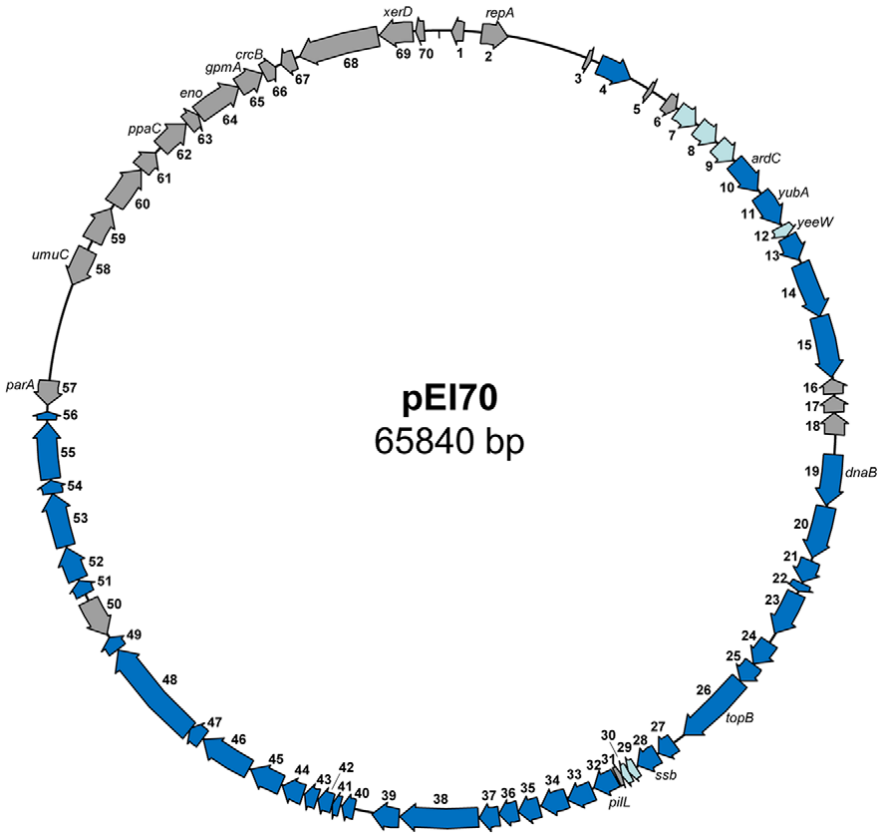
Circular representation of plasmid pEI70. Locus tags (EaACW_pEI700xx) are indicated in the graph. Genes in dark blue are genes with orthologs in ICE elements. Genes that were also identified in the ICE element of *E. pyrifoliae*, but which are not related to a function in the ICE element are highlighted in light blue. Genes in grey are unrelated to the ICE element.

*E. amylovora* strain IVIA 1614-2a was previously shown to contain an approximately 70 kb plasmid and to lack the ubiquitous plasmid pEA29 [24]. The sequencing of the plasmid yielded a final consensus sequence of 65,840 base pairs, with an overall G+C content of 52.2%, close to the G+C level of the host [13], [34]. A total of 70 CDS were identified by GenDB and manually annotated (Fig. 1).

### Sequence analysis of pEI70

**Figure 2:**
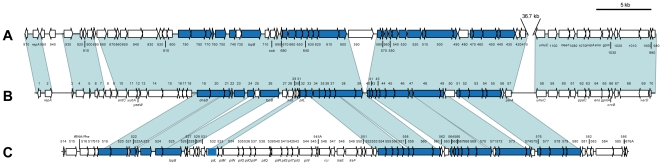
Comparison of plasmid pEB102 of *E. billingiae* Eb661 (A) with *E. amylovora* ACW 56400 plasmid pEI70 (B), and the conserved region of GAI-2 of *Pectobacterium atrosepticum* SCRI 1043 (C). Orthologous genes are indicated by blue shading (conserved ICE element genes) and shading. Genes in white do not have orthologs in these regions.

Of the 70 CDS identified, 61 have more than 98% sequence identity to sequences of plasmid pEB102 from *E. billingiae* strain Eb661 (see Table S1). The initiator replication protein RepA has 99% sequence identity to the RepA of plasmid pEB102 and a 98% identity to plasmid pPATH from *Pantoea agglomerans* pv. *gypsophilae* 824-1 [35], indicating a potential common origin of these three plasmids. The nucleotide sequences of genes encoded on pEI70 have more than 98% identity to their counterparts on plasmid pEB102, as observed by BLASTN analysis. The organization of the CDS in plasmids pEI70 and pEB102 are identical as well, but a 36-kb region in pEB102 is absent in pEI70 (Fig. 2). In this region, genes encoding proteins putatively involved in LPS biosynthesis (EbC_pEb10200120-130) and resistance to arsenate (EbC_pEb10200180-210) are present among many others with no or only a general function prediction [36]. A major feature of pEI70 is the presence of an Integrating Conjugative Element (ICE) that shares similarities to regions of PFGI-01 of *Pseudomonas fluorescens* Pf-5 [37] and HAI2 of *Pectobacterium atrosepticum* SCRI 1043 [38], containing a fragment of the *pilL* gene and lacks all the *tra* genes (Fig. 2C). We were not able to find potential attenuation sites [39] in the sequence of pEI70 that would allow an insertion into or close to known insertion sites in the genome of *E. amylovora* CFBP 1430 [13], [39]. This would indicate that the ICE might be unable to integrate into the chromosome and therefore, remains only present as a plasmid in *E. amylovora* strains.

### Plasmid stability and conjugability of pEI70

We evaluated the stability of pEI70 in two native host strains: one with only this plasmid (strain IVIA 1614-2a) and another harboring pEA29 and pEI70 (strain IVIA 1614-1) in a 200-generation analysis. A total of 100 colonies per strain were positive by PCR for the presence of the plasmid after the 200-generations transfer in each of the two analyses performed. All the plasmid extractions performed (25 colonies) demonstrated that the plasmid was not integrated into the chromosome of the bacterium and that the plasmid did not change over time. Plasmid maintenance of pEI70 was 100% after 200 generations, both in the presence and absence of pEA29, and this latter plasmid was also found in 100% of the colonies analyzed. These experiments indicated that both plasmids are highly stable in their native *E. amylovora* host strains, and also the high stability of the pEI70 even in the presence of pEA29.

Conjugation experiments showed that pEI70 can be transferred between *E. amylovora* strains. A Tn5-tagged plasmid pEI70::Tn5 was conjugatively transferred into the strain CGJ2 that already contained the transposon-tagged plasmid pEA29::Tn5393, resulting in a trans-conjugant with both plasmids and two antibiotic resistances. The possibility that the trans-conjugants containing both plasmids arose from the mobilization of pEA29::Tn5393 into the strain containing pEI70 is quite unlikely, as the former plasmid was not shown to be conjugative [40] and the analysis of plasmid content showed that no plasmids were present in strain CGJ2.

### Distribution of pEI70 within isolates from geographically different regions

**Figure 3:**
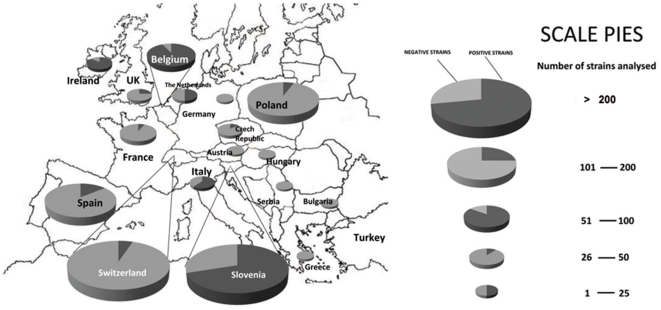
Biogeographic distribution map of strains harboring pEI70 in different European countries obtained by duplex-PCR.

The results obtained from the duplex PCR that allows the simultaneous detection of plasmids pEA29 and pEI70 in the different strains analyzed are shown in Table 3 and Fig. 3. Additional information on the strains positive for pEI70 analyzed, is shown in Table S2. Plasmid pEI70 was found to be widespread throughout European countries. In total, this new plasmid was found in 458 strains out of 1,249 isolates analyzed from Europe (36.6%). The proportion of strains containing pEI70 plasmid was variable, ranging from 5% to 92% of the European strains analyzed, depending on the country (Table 3). Prevalence was as high as 62.9%, 85% and 92% in Slovenia, Ireland, and Belgium, respectively. In some countries (i.e., Austria, Bulgaria, Germany, Greece and Hungary), no strains containing pEI70 were detected, but in several of these countries, the number of isolates analyzed was quite low. Additional data on the strains negative for pEI70 analyzed is shown in Table S3. In other countries (i.e., Czech Republic, Italy and The Netherlands), at least one strain was found with plasmid pEI70, even when only a few strains were analyzed. In Spain, from 142 strains obtained in several outbreaks from 1995 to 2009, 20 strains (14%) were found carrying pEI70, and of these, three were also devoid of pEA29. The information from supplementary Table S2 demonstrated that at least since 1972 the plasmid was present in a French strain and since 1979 in another from Belgium. It is interesting to remark that more than twenty years later, strains with this plasmid were still isolated in Belgium confirming the natural stability of this plasmid in wild *E. amylovora* strains, which has been observed in our *in vitro* assays. Of the 231 strains analyzed from countries of other continents (e.g., Canada, Lebanon, Turkey, USA), pEI70 has thus far not been detected even in the USA, where fire blight originated.

**Figure 4:**
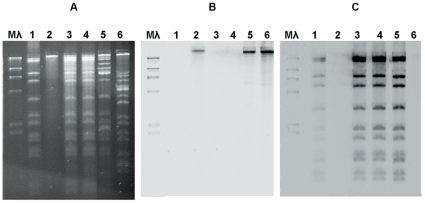
Analysis of several strains by restriction digestion with *Bam*HI (A) and hybridisation analyses using pEA29 as probe (B) or pEI70 as probe (C). Isolate E 70 (lane 5) from Ireland shows a slightly different profile, with two extra bands that do not belong either to pEA29 or pEI70. Lane 1: strain IVIA 1614-2a; lane 2: CFBP 1430; lane 3: IVIA 1596; lane 4: NCPPB 3299; lane 5: E70; lane 6: Ea 273. M λ: marker Lambda (Invitrogen).

The presence of pEI70 was confirmed by plasmid extraction and restriction with the *Bam*HI enzyme in 13 strains (eight from Poland, three from Spain, one from UK and one from Ireland), all providing the same restriction pattern. The strain from Ireland (E70) harbored another plasmid of around 30 kb, different to pEA29, as observed after restriction analyses and hybridization with pEA29 as a probe (Fig. 4).

Only six strains (three from Spain, two from Serbia and one from Belgium) were found not to carry plasmids (0.5%).

### Analyses of aggressiveness using *ex vivo* plant assays

**Figure 5:**
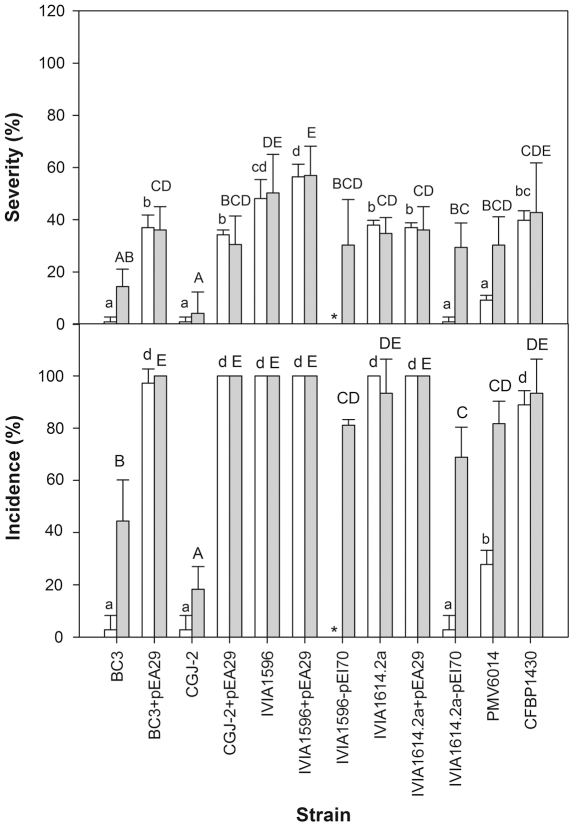
Severity and infection incidence in immature pear fruits by inoculation of strains of *E. amylovora* before and after receiving plasmid pEA29 or after curing of pEI70. The severity and incidence of infection were measured 5 days after inoculation. The experiment was performed at 1×10^5^ cfu/ml (white columns) and 1×10^6^ cfu/ml (black columns). Means with the same letters (lower-case letters for low pathogen dosages, upper-case letters for high pathogen dosages) do not differ significantly according to Tukey's test (*P*≤0.05). Asterisks ‘*’ indicate assays that were not performed.

The role of pEA29 and pEI70 on the aggressiveness of *E. amylovora* strains was tested in immature pear fruits. In the first assay, large differences in aggressiveness between strains with different plasmid contents were observed (Table 4; Fig. 5). Plasmid-less strains CGJ2 and BC3 showed a very low level of aggressiveness, similar to the strain PMV 6014 (strain CFBP 1430 cured of pEA29); whereas the most aggressive strains contained at least one of the plasmids. When comparing the strains before and after introducing pEA29 it was observed that the presence of this plasmid significantly increased the incidence and severity of infections in the least aggressiveness strains (i.e., BC3, CGJ-2 and PMV 6014), independently of the inoculation dose. However, differences were more evident when strains were inoculated at a lower dose (10^5^ cfu/ml). The strains cured of pEI70 (i.e., IVIA 1596-pEI70 and IVIA 1614-2a-pEI70) caused significantly less infection incidence and lower severity compared to the wild type, and again this was more evident with a lower dose of inoculation (Fig. 5).

**Figure 6:**
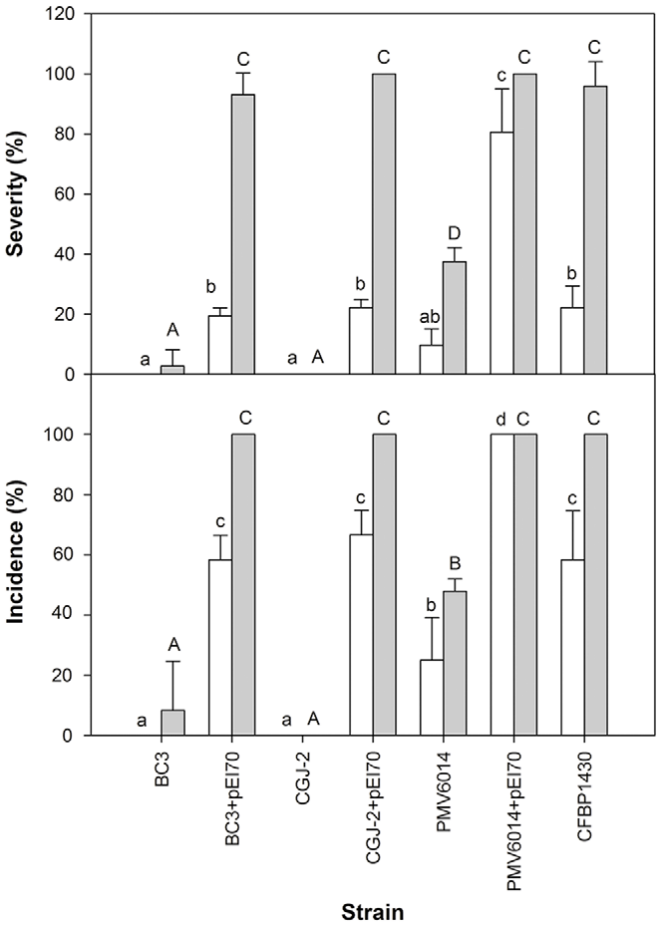
Severity and infection incidence in immature pear fruits by inoculation of strains of *E. amylovora* with or without pEI70. The severity and incidence of infection were measured 7 days after inoculation. The experiment was performed at 1×10^5^ cfu/ml (white columns) and 5×10^5^ cfu/ml (black columns). Means with the same letters (lower-case letters for low dosages, upper-case letters for high dosages) do not differ significantly according to Tukey's test (*P*≤0.05).

In the second assay, an increase in aggressiveness was also observed when pEI70 was introduced in strains that did not contain this plasmid (Fig. 6). This increase was observed at both doses assessed and was similar to the increase observed after the introduction of pEA29 in the strains that showed lower levels of aggressiveness.

Therefore, we can conclude that pEI70 has a clear effect on the aggressiveness of these strains (Table 4, Fig. 5, Fig. 6).

## Discussion

Several studies on the aggressiveness and phenotypic characteristics of *E. amylovora* strains have shown different levels of variability among them, regardless of the homogeneous genetic content observed in this species [11], [13]. An important source of genetic variability comes from gaining new genes through plasmid acquisition, as observed in other bacterial models [41]–[44]. In *E. amylovora*, some studies have been performed on the plasmid content of the strains and the functions they could provide to the bacterial genome, but these have been very limited in terms of the number of strains analyzed. Until now, apart from the plasmid pEA29 and its functionality in virulence, no recent data are available [16], [17], [45]. The detection of wild isolates lacking pEA29, and the presence of plasmid pEI70 that some of these strains instead carry [24], [46] has prompted a detailed analysis of this new plasmid and the elucidation of its role in the interaction between bacterium and plant host.

Analysis of the pEI70 sequence showed a very high sequence identity (more than 98% identity) to plasmid pEB102 from *E. billingiae* Eb661, which suggests a common origin of both plasmids. Plasmid pEI70 presumably encodes an ICE, but lacks the Type IV Secretion System (T4SS) *tra* and *pil* genes. Their gene products are commonly known to be involved in conjugative transfer, but they are not present in all known ICEs [47], [48]. Nevertheless, plasmid pEI70 is conjugatively transferred to other *E. amylovora* strains, disputing the role of T4SS in the conjugative transfer of this kind of mobile element.

The genes in the latter quarter of the plasmid sequence do not belong to the core of the ICE, and may represent the “cargo” genes. The annotation predicts a metabolic function for several of these genes, which would suggest that these genes optimize the performance of the pathways they are involved in. The *eno* and *gpmA* genes (enolase and phosphoglyceromutase) present in the plasmid are enzymes of the glycolysis pathway. In preliminary experiments, it was observed that the growth rates of several strains assayed in minimal medium with sucrose were significantly faster in strains with pEI70, compared to the same strains lacking the plasmid. This effect was not observed when these strains were grown in rich medium like LB, which would indicate that the glycolysis genes may play a role (data not shown). *In planta*, this may improve competence for degrading these compounds, increasing pathogen aggressiveness. We hypothesize that the glycolysis in *E. amylovora* may be one of the bottlenecks in the bacterial growth and subsequent infection *in planta*. The fact that these genes are almost identical in plasmid pEB102 suggests a fitness effect rather than a role in virulence because the effect it provides in the epiphytic *E. billingiae* would likely confer a metabolic advantage. The relatedness of plasmids among closely related *Erwinia* species suggests occurrence of horizontal genetic transfer.

An interesting attribute of pEI70 is that it seems to be widespread in Europe because it has been detected in strains from 11 countries. The screening of 1,249 strains from 17 countries for the presence of this new plasmid has shown that it can be very abundant in some Western, Central and North-eastern European countries, but it is absent in isolates from the South-eastern European countries analyzed, or in countries outside the European continent. In Belgium, the percentage of strains positive for pEI70 was very high (more than 90% of isolates analyzed) but some distant countries also show high percentages (e.g., Slovenia, 62.9%). For instance, in Spain, pEI70 was present in 14% of the strains analyzed and in 6 out of the 9 regions where the disease was reported up to 2009. In other European countries, plasmid pEI70 was not detected (e.g., Austria and Germany), but the low number of strains analyzed and proximity to countries with high prevalence of plasmid pEI70 (Fig. 3) suggest that further sampling will probably reveal its presence. More complete analyses could provide some useful information about the dispersal of the disease in Europe, although it is necessary to analyze more strains from the remaining countries and increase the number of isolates in some others to obtain solid conclusions (Tables S2 and S3).

Although a large number of strains from North America were examined, plasmid pEI70 was not detected, even though the disease originated in the USA [2], suggesting that this plasmid maybe was introduced once *E. amylovora* spread to Europe. Other regions where fire blight occurs (e.g., Northern Africa, New Zealand and Mexico) remain to be surveyed using the primer sets described in this paper.

The analyses on immature pear fruit have shown that, when pEI70 was introduced into strains with low levels of aggressiveness, the intensity of symptoms increased. Moreover, after curing this plasmid from two strains (i.e., IVIA 1596 and IVIA 1614-2a) that show a standard level of aggressiveness, the intensity of symptoms decreased to levels similar to the strains without plasmids or to the strain PMV 6014, cured of pEA29. However, all strains tested containing only pEI70 had a standard to high level of aggressiveness. In preliminary experiments, the analyses of strains devoid of plasmids showed the same variability in aggressiveness as typical *E. amylovora* strains harboring pEA29: we found strains with high aggressiveness (e.g., UPN527 from Spain) and also strains with low aggressiveness (e.g., BC3 and CGJ2 from Serbia). Wild-type strains lacking pEA29, but containing plasmid pEI70, presented similar levels of aggressiveness compared to the reference strain CFBP1430, that harbors only pEA29 (Fig. 5). In contrast, no significant effect of the introduction of pEA29 was observed on the incidence of infection in two strains that naturally only harbor plasmid pEI70 (i.e., IVIA 1596 and IVIA 1614-2a) (Table 4). In other studies, introduction of pEA29 in strains without this plasmid provided different levels of aggressiveness, depending on the strain studied [49]. These results suggest the possible influence of other factors such as genomic background, host interactions and environmental conditions on the variability in aggressiveness that remains unexplained. In recent years, several *E. amylovora* strains with differing plasmid contents, including strains without pEA29 or strains with new plasmids, have been discovered [16]–[24]. The results obtained on symptom development after their inoculation suggest that variable genomic backgrounds could be behind the differences in aggressiveness of *E. amylovora* strains, and that still unknown mechanisms may play a role in its aggressiveness, including a possible effect of the plasmid genes on the expression of chromosomal genes. Bacterial plasmids can contain so-called “fitness island” sequences (FIs), which consist of genes responsible for the epiphytic fitness of the bacterium that could enhance their pathogenicity and virulence as an additional advantage [42], [43], [45]. Plasmid pEI70 seems to provide some features that compensate for the lack of pEA29 and could explain the standard aggressiveness levels observed in the strains harboring it. Recently, in *E. amylovora* strains from Poland a new plasmid has been found (J. Pulawska, *personal communication*), which indicates the need for additional detailed studies on the only factor currently known to influence the pan-genome in this important plant pathogen [14]. Most likely, different plasmids are still waiting to be discovered in *E. amylovora* strains, and further studies at genomic, proteomic and metabolomic levels could contribute to fully understanding the role of extra-genetic elements in the *E. amylovora*-plant interaction.

## Supporting Information

Table S1 Predicted CDS to proteins of pEI70 in the GenBank nonredundant database.
(DOC)

Table S2 Additional information available from *E. amylovora* strains positive for pEI70 analyzed in European countries.
(DOC)

Table S3 Additional information available from *E. amylovora* strains negative for pEI70 analyzed in European countries.
(PDF)
