# Supplementary material for: Erwinia amylovora Novel Plasmid pEI70: Complete Sequence, Biogeography, and Role in Aggressiveness in the Fire Blight Phytopathogen
Source: PLoS One. 2011 Dec 9;6(12):e28651. doi: 10.1371/journal.pone.0028651 (PMC3235134; doi:10.1371/journal.pone.0028651)
Supplement: Table S1 — Predicted CDS to proteins of pEI70 in the GenBank nonredundant database. (DOC) [file pone.0028651.s001.doc]

**Table S1**.

| **Locus** | **Gene** | **Best BlastP hit** | **% amino acid** | **Accession number** |
| --- | --- | --- | --- | --- |
| **tag** | **name** | **Sequence identity** | **identity** |  |
| 01 |  | ArdK protein *E. billingiae* Eb661 plasmid pEB102 | 99 | [YP_003739154.1](http://www.ncbi.nlm.nih.gov/protein/300781919?report=genbank&log$=prottop&blast_rank=1&RID=2JN18BX601S) |
| 02 | *repA* | RepA replication initiation protein E. billingiae Eb661 plasmid pEB102 | 99 | YP_003739153.1 |
| 03 |  | No significant similarity found | - | - |
| 04 |  | Integrase E. billingiae Eb661 plasmid pEB102 | 99 | YP_003739150.1 |
| 05 |  | No significant similarity found | - | - |
| 06 |  | Hypothetical protein *E. billingiae* Eb661 plasmid pEB102 | 99 | YP_003739148 |
| 07 |  | Hypothetical protein *E. billingiae* Eb661 plasmid pEB102 | 99 | [YP_003739147.1](http://www.ncbi.nlm.nih.gov/protein/300781912?report=genbank&log$=prottop&blast_rank=1&RID=2JVJJYH4016) |
| 08 |  | Hypothetical protein *Escherichia coli* UMNK88 | 33 | AEE59532 |
| 09 |  | Hypothetical protein *Serratia* sp. AS12 | 31 | YP_004498793.1 |
| 10 | *ardC* | Antirestriction protein ArdC *E. billingiae* Eb661 plasmid pEB102 | 99 | YP_003739145.1 |
| 11 | *yubA* | Protein of unknown function (DUF1281) *E. billingiae* Eb661 plasmid pEB102 | 99 | YP_003739144.1 |
| 12 | *yeeW* | Hypothetical protein *E. billingiae* Eb661 plasmid pEB102 | 99 | YP_003739143.1 |
| 13 |  | Hypothetical protein *E. billingiae* Eb661 plasmid pEB102 | 99 | YP_003739142.1 |
| 14 |  | DNA helicase *E. billingiae* Eb661 plasmid pEB102 | 100 | YP_003739141.1 |
| 15 |  | Putative helicase/relaxase *E. billingiae* Eb661 plasmid pEB102 | 99 | YP_003739140.1 |
| 16 |  | Predicted thioesterase *E. billingiae* Eb661 plasmid pEB102 | 100 | YP_003739139.1 |
| 17 |  | Hypothetical protein *E. billingiae* Eb661 plasmid pEB102 | 99 | YP_003739138.1 |
| 18 |  | Hypothetical protein *E. billingiae* Eb661 plasmid pEB102 | 99 | YP_003739137.1 |
| 19 | *dnaB* | Replicative DNA helicase  *E. billingiae* Eb661 plasmid pEB102 | 99 | [YP_003739136.1](http://www.ncbi.nlm.nih.gov/protein/300781901?report=genbank&log$=prottop&blast_rank=1&RID=2UBX4GC001N) |
| 20 |  | ICE-PFGI_1_parB, ParB family protein *E. billingiae* Eb661 plasmid pEB102 | 99 | [YP_003739135.1](http://www.ncbi.nlm.nih.gov/protein/300781900?report=genbank&log$=prottop&blast_rank=1&RID=2UCJKBP001N) |
| 21 |  | DUF2857 superfamily protein *E. billingiae* Eb661 plasmid pEB102 | 100 | YP_003739134.1 |
| 22 |  | Conserved hypothetical protein *E. billingiae* Eb661 plasmid pEB102 | 100 | YP_003739133.1 |
| 23 |  | Conserved hypothetical protein *E. billingiae* Eb661 plasmid pEB102 | 99 | YP_003739132.1 |
| 24 |  | Integrating conjugative element protein, PFL_4669 family *E. billingiae* Eb661 plasmid pEB102 | 100 | YP_003739131.1 |
| 25 |  | Conserved hypothetical protein *E. billingiae* Eb661 plasmid pEB102 | 100 | [YP_003739130.1](http://www.ncbi.nlm.nih.gov/protein/300781895?report=genbank&log$=prottop&blast_rank=1&RID=2UDRH9RN012) |
| 26 | *topB* | DNA topoisomerase III *E. billingiae* Eb661 plasmid pEB102 | 99 | YP_003739129.1 |
| 27 |  | Conserved hypothetical protein *E. billingiae* Eb661 plasmid pEB102 | 100 | YP_003739128.1 |
| 28 | *ssb* | Single-stranded DNA-binding protein *E. billingiae* Eb661 plasmid pEB102 | 99 | YP_003739127.1 |
| 29 |  | Conserved hypothetical protein *E. billingiae* Eb661 plasmid pEB102 | 99 | YP_003739126.1 |
| 30 |  | Hypothetical protein *Serratia symbiotica* str. Tucson | 76 | ZP_08039662.1 |
| 31 |  | Hypothetical protein *E. billingiae* Eb661 plasmid pEB102 | 100 | YP_003739125.1 |
| 32 | *pilL* | Similar to type IV B pilus protein *E. billingiae* Eb661 plasmid pEB102 | 100 | YP_003739124.1 |
| 33 |  | Hypothetical protein *E. billingiae* Eb661 plasmid pEB102 | 99 | YP_003739123.1 |
| 34 |  | ICE protein, PFL_4693 family *E. billingiae* Eb661 plasmid pEB102 | 99 | YP_003739122.1 |
| 35 |  | Putative lytic transglycosylase, catalytic *E. billingiae* Eb661 plasmid pEB102 | 100 | YP_003739121.1 |
| 36 |  | ICE protein, PFL_4695 family *E. billingiae* Eb661 plasmid pEB102 | 100 | YP_003739120.1 |
| 37 |  | Restriction endonuclease, type IV-like *E. billingiae* Eb661 plasmid pEB102 | 100 | YP_003739119.1 |
| 38 |  | Conjugative coupling factor TraD, SXT/TOL subfamily *E. billingiae* Eb661 plasmid pEB102 | 100 | YP_003739118.1 |
| 39 |  | ICE membrane protein, PFL_4697 family *E. billingiae* Eb661 plasmid pEB102 | 99 | YP_003739117.1 |
| 40 |  | Plasmid conserved hypothetical protein, RAQPRD family *E. billingiae* Eb661 plasmid pEB102 | 98 | YP_003739115.1 |
| 41 |  | ICE protein, PFL_4701 family*E. billingiae* Eb661 plasmid pEB102 | 100 | YP_003739114.1 |
| 42 |  | ICE membrane protein, PFL_4702 family *E. billingiae* Eb661 plasmid pEB102 | 99 | YP_003739113.1 |
| 43 |  | Conjugative transfer region protein, TIGR03750 family *E. billingiae* Eb661 plasmid pEB102 | 100 | YP_003739112.1 |
| 44 |  | ICE protein, PFL_4703 family *E. billingiae* Eb661 plasmid pEB102 | 99 | YP_003739111.1 |
| 45 |  | ICE protein, PFL_4704 family *E. billingiae* Eb661 plasmid pEB102 | 98 | YP_003739110.1 |
| 46 |  | ICE protein, PFL_4705 family *E. billingiae* Eb661 plasmid pEB102 | 99 | [YP_003739109.1](http://www.ncbi.nlm.nih.gov/protein/300781874?report=genbank&log$=prottop&blast_rank=1&RID=2UM8KTY3012) |
| 47 |  | Conjugative transfer region lipoprotein, TIGR03751 family *E. billingiae* Eb661 plasmid pEB102 | 99 | YP_003739108.1 |
| 48 |  | Putative plasmid-related outer membrane ATPase *E. billingiae* Eb661 plasmid pEB102 | 99 | YP_003739107.1 |
| 49 |  | Conjugative transfer ATPase, PFL_4706 family *E. billingiae* Eb661 plasmid pEB102 | 98 | YP_003739106.1 |
| 50 |  | Hypothetical protein *E. billingiae* Eb661 plasmid pEB102 | 99 | YP_003739105.1 |
| 51 |  | Conserved hypothetical protein *E. billingiae* Eb661 plasmid pEB102 | 100 | YP_003739104.1 |
| 52 |  | ICE protein, PFL_4709 family *E. billingiae* Eb661 plasmid pEB102 | 99 | YP_003739103.1 |
| 53 |  | ICE protein, PFL_4710 family *E. billingiae* Eb661 plasmid pEB102 | 100 | YP_003739102.1 |
| 54 |  | ICE protein, PFL_4711 family *E. billingiae* Eb661 plasmid pEB102 | 99 | YP_003739101.1 |
| 55 |  | Conserved hypothetical protein *E. billingiae* Eb661 plasmid pEB102 | 99 | YP_003739100.1 |
| 56 |  | TraG_N superfamily protein *Burkholderia gladioli* BSR3 chromosome 2 | 26 | YP_004349864.1 |
| 57 | *parA* | Putative site-specific recombinase, resolvase family *E. billingiae* Eb661 plasmid pEB102 | 98 | YP_003739098.1 |
| 58 | *umuC* | DNA-repair protein, UmuC-like *Pantoea* sp. At-9b plasmid pPAT9B05 | 83 | YP_004119580.1 |
| 59 |  | WD40 superfamily protein *E. billingiae* Eb661 plasmid pEB102 | 100 | YP_003739166.1 |
| 60 | *mae1* | C4-dicarboxylate transporter/malic acid transport protein *E. billingiae* Eb661 plasmid pEB102 | 100 | YP_003739165.1 |
| 61 |  | Uracil-DNA glycosylase family protein *E. billingiae* Eb661 plasmid pEB102 | 100 | YP_003739164.1 |
| 62 | *ppaC* | Inorganic pyrophosphatase *E. billingiae* Eb661 plasmid pEB102 | 100 | YP_003739163.1 |
| 63 |  | Universal stress protein A  *E. billingiae* Eb661 plasmid pEB102 | 100 | YP_003739162.1 |
| 64 | *eno* | Enolase *E. billingiae* Eb661 plasmid pEB102 | 100 | [YP_003739161.1](http://www.ncbi.nlm.nih.gov/protein/300781926?report=genbank&log$=protalign&blast_rank=1&RID=2UT7A7YJ01N) |
| 65 | *gpmA* | 3-bisphosphoglycerate-dependent phosphoglycerate mutase *Pantoea ananatis* AJ13355 | 92 | [BAK12605.1](http://www.ncbi.nlm.nih.gov/protein/327395183?report=genbank&log$=prottop&blast_rank=1&RID=2UTH5HR0016) |
| 66 | *crcB* | Camphor resistance CrcB family protein  *E. billingiae* Eb661 plasmid pEB102 | 100 | YP_003739159.1 |
| 67 |  | Glyoxalase/bleomycin resistance protein/dioxygenase *E. billingiae* Eb661 plasmid pEB102 | 100 | YP_003739158.1 |
| 68 |  | Site-specific recombinase, phage integrase family  *E. billingiae* Eb661 plasmid pEB102 | 100 | YP_003739157.1 |
| 69 | *xerD* | Tyrosine recombinase xerC *E. billingiae* Eb661 plasmid pEB102 | 100 | YP_003739156.1 |
| 70 | *samB* | DNA polymerase IV.DNA-directed DNA polymerase *Pantoea* sp. At-9b plasmid pPAT9B05 | 79 | YP_004119580.1 |
