# Supplementary material for: Erwinia amylovora Novel Plasmid pEI70: Complete Sequence, Biogeography, and Role in Aggressiveness in the Fire Blight Phytopathogen
Source: PLoS One. 2011 Dec 9;6(12):e28651. doi: 10.1371/journal.pone.0028651 (PMC3235134; doi:10.1371/journal.pone.0028651)
Supplement: Table S2 — Additional information available from E. amylovora strains positive for pEI70 analyzed in European countries. (DOC) [file pone.0028651.s002.doc]

Table S2.

| **Country** | **Collection** | **Isolates positive for pEI70** | **Host** | **Year of isolation** |
| --- | --- | --- | --- | --- |
| Belgium | LMG | LMG 1880 | Pear | 1979 |
| Belgium | LMG | LMG 1881 | *Crataegus* sp. | 1980 |
| Belgium | LMG | LMG 1882 | Pear | 1977 |
| Belgium | LMG | LMG 1883 | Pear | 1979 |
| Belgium | LMG | LMG 1885 | Crataegus sp. | 1980 |
| Belgium | LMG | LMG 1889 | *Crataegus* sp. | 1980 |
| Belgium | LMG | LMG 1892 | *Cotoneaster* sp. | 1980 |
| Belgium | LMG | LMG 1893 | Pear | 1980 |
| Belgium | LMG | LMG 1895 | *Crataegus* sp. | 1980 |
| Belgium | LMG | LMG 1899 | Pear | 1980 |
| Belgium | LMG | LMG 1906 | *Crataegus* sp. | 1980 |
| Belgium | LMG | LMG 1907 | Pear | 1980 |
| Belgium | LMG | LMG 1910 | Pear | 1980 |
| Belgium | LMG | LMG 1911 | *Cotoneaster* sp. | 1980 |
| Belgium | LMG | LMG 1931 | Pear | 1980 |
| Belgium | LMG | LMG 1932 | *Cotoneaster* sp. | 1980 |
| Belgium | LMG | LMG 1933 | *Cotoneaster* sp. | 1980 |
| Belgium | LMG | LMG 1934 | Pear | 1980 |
| Belgium | LMG | LMG 1936 | *Crataegus* sp. | 1980 |
| Belgium | LMG | LMG 1937 | *Cotoneaster* sp. | 1980 |
| Belgium | LMG | LMG 1946 | Pear | 1980 |
| Belgium | LMG | LMG 1947 | *Crataegus* sp. | 1980 |
| Belgium | LMG | LMG 1955 | *Crataegus* sp. | 1980 |
| Belgium | LMG | LMG 1961 | Crataegus sp. | 1980 |
| Belgium | CRA-W | EaP1 | Pear | 2001 |
| Belgium | CRA-W | EaP2 | Pear | 2001 |
| Belgium | CRA-W | EaP5 | Pear | 2001 |
| Belgium | CRA-W | EaP18 | Pear | 2001 |
| Belgium | CRA-W | EaP24 | Pear | 2001 |
| Belgium | CRA-W | EaP25 | Pear | 2001 |
| Belgium | CRA-W | EaP26 | Pear | 2001 |
| Belgium | CRA-W | EaP27 | Pear | 2001 |
| Belgium | CRA-W | EaP28 | Pear | 2001 |
| Belgium | CRA-W | EaP29 | Pear | 2002 |
| Belgium | CRA-W | EaP30 | Pear | 2002 |
| Belgium | CRA-W | EaP31 | Pear | 2002 |
| Belgium | CRA-W | EaP32 | Pear | 2002 |
| Belgium | CRA-W | EaP33 | Pear | 2002 |
| Belgium | CRA-W | EaP34 | Pear | 2002 |
| Belgium | CRA-W | EaP35 | Pear | 2002 |
| Belgium | CRA-W | EaP36 | Pear | 2002 |
| Belgium | CRA-W | EaP38 | Pear | 2002 |
| Belgium | CRA-W | EaP39 | Pear | 2002 |
| Belgium | CRA-W | EaP40 | Pear | 2002 |
| Belgium | CRA-W | EaP41 | Pear | 2002 |
| Belgium | CRA-W | EaP42 | Pear | 2002 |
| Belgium | CRA-W | EaP43 | Pear | 2002 |
| Belgium | CRA-W | EaP44 | Pear | 2002 |
| Belgium | CRA-W | EaP46 | Pear | 2002 |
| Belgium | CRA-W | EaP47 | Pear | 2002 |
| Belgium | CRA-W | EaP48 | Pear | 2002 |
| Belgium | CRA-W | EaP49 | Pear | 2002 |
| Belgium | CRA-W | EaP50 | Pear | 2002 |
| Belgium | CRA-W | EaP51 | Pear | 2002 |
| Belgium | CRA-W | EaP52 | Pear | 2002 |
| Belgium | CRA-W | EaP53 | Pear | 2004 |
| Belgium | CRA-W | EaA9 | Crataegus sp. | 2001 |
| Belgium | CRA-W | EaA10 | *Crataegus* sp. | 2001 |
| Belgium | CRA-W | EaA11 | *Crataegus* sp. | 2001 |
| Belgium | CRA-W | EaA12 | *Crataegus* sp. | 2001 |
| Belgium | CRA-W | EaA14 | *Crataegus* sp. | 2001 |
| Belgium | CRA-W | EaA15 | *Crataegus* sp. | 2001 |
| Belgium | CRA-W | EaA17 | *Crataegus* sp. | 2001 |
| Belgium | CRA-W | EaA20 | *Crataegus* sp. | 2001 |
| Belgium | CRA-W | EaA54 | *Crataegus* sp. | 2001 |
| Czech Republic | BPIC | BPIC 1634 | Sorbus sp. | 1990 |
| France | CFBP | CFBP1367 | *Crataegus* sp. | 1972 |
| France | CFBP | CFBP1368 | Pear | 1972 |
| France | LNPV | LNPV 1585 | *Cotoneaster* sp. | na |
| Ireland | SL | SL 1014 | *Pyracantha* sp. | 1986 |
| Ireland | SL | SL 2156 | *Cotoneaster* sp. | na |
| Ireland | SL | SL 2157 | *Cotoneaster* sp. | na |
| Ireland | SL | SL 2158 | *Sorbus* sp. | na |
| Ireland | SL | SL 2159 | *Sorbus* sp. | na |
| Ireland | SL | SL 2160 | *Sorbus* sp. | na |
| Ireland | SL | SL 2161 | *Sorbus* sp. | na |
| Ireland | SL | SL 2162 | *Sorbus* sp. | na |
| Ireland | SL | SL 2163 | *Cotoneaster* sp. | na |
| Ireland | SL | SL 2164 | *Sorbus* sp. | na |
| Ireland | CFBP | CFBP 2584 | *Cotoneaster* sp. | 1986 |
| Ireland | DERM | E-70 | *Cotoneaster* sp. | 1997 |
| Italy | OMP | OMP-BO 1185 | Apple | 1997 |
| Italy | SFR | SFR-BO 685 | Crataegus sp. | 1999 |
| Italy | SFR | SFR- BO 984 | Crataegus sp. | 1999 |
| Poland | DPP | 2 | *Crataegus* sp. | 2000 |
| Poland | DPP | 600 | *Crataegus* sp. | 1993 |
| Poland | DPP | 601 | Pear | 1993 |
| Poland | DPP | 603 | Crataegus sp. | 1994 |
| Poland | DPP | 606 | *Crataegus* sp. | 1994 |
| Poland | DPP | 665 | Pear | 1993 |
| Poland | DPP | 666 | *Crataegus* sp. | 1996 |
| Slovenia | NIB | 109 | *Cydonia* sp. | 2002 |
| Slovenia | NIB | 110 | *Cydonia* sp. | 2002 |
| Slovenia | NIB | 114 | *Cydonia* sp. | 2002 |
| Slovenia | NIB | 115 | *Cydonia* sp. | 2002 |
| Slovenia | NIB | 170 | *Cydonia* sp. | 2003 |
| Slovenia | NIB | 171 | Pear | 2003 |
| Slovenia | NIB | 172 | *Cydonia* sp. | 2003 |
| Slovenia | NIB | 173 | Apple | 2003 |
| Slovenia | NIB | 175 | Apple | 2003 |
| Slovenia | NIB | 176 | *Cydonia* sp. | 2003 |
| Slovenia | NIB | 177 | Apple | 2003 |
| Slovenia | NIB | 178 | *Pyracantha* sp. | 2003 |
| Slovenia | NIB | 180 | *Pyracantha* sp. | 2003 |
| Slovenia | NIB | 181 | Apple | 2003 |
| Slovenia | NIB | 182 | Pear | 2003 |
| Slovenia | NIB | 183 | *Cotoneaster* sp. | 2003 |
| Slovenia | NIB | 185 | Apple | 2003 |
| Slovenia | NIB | 189 | *Crataegus* sp. | 2003 |
| Slovenia | NIB | 190 | Apple | 2003 |
| Slovenia | NIB | 191 | *Cydonia* sp. | 2003 |
| Slovenia | NIB | 192 | Pear | 2003 |
| Slovenia | NIB | 193 | Apple | 2003 |
| Slovenia | NIB | 195 | Apple | 2003 |
| Slovenia | NIB | 199 | Pear | 2003 |
| Slovenia | NIB | 202 | Pear | 2003 |
| Slovenia | NIB | 203 | Apple | 2003 |
| Slovenia | NIB | 204 | Pear | 2003 |
| Slovenia | NIB | 205 | Apple | 2003 |
| Slovenia | NIB | 206 | Apple | 2003 |
| Slovenia | NIB | 207 | Apple | 2003 |
| Slovenia | NIB | 208 | Apple | 2003 |
| Slovenia | NIB | 210 | Apple | 2003 |
| Slovenia | NIB | 211 | Apple | 2003 |
| Slovenia | NIB | 212 | Apple | 2003 |
| Slovenia | NIB | 213 | Apple | 2003 |
| Slovenia | NIB | 215 | Apple | 2003 |
| Slovenia | NIB | 216 | Apple | 2003 |
| Slovenia | NIB | 217 | Pear | 2003 |
| Slovenia | NIB | 218 | Apple | 2003 |
| Slovenia | NIB | 219 | Apple | 2003 |
| Slovenia | NIB | 224 | Apple | 2003 |
| Slovenia | NIB | 225 | Apple | 2003 |
| Slovenia | NIB | 226 | Apple | 2003 |
| Slovenia | NIB | 228 | *Pyracantha* sp. | 2003 |
| Slovenia | NIB | 231 | Apple | 2003 |
| Slovenia | NIB | 235 | Pear | 2003 |
| Slovenia | NIB | 236 | Apple | 2003 |
| Slovenia | NIB | 237 | Apple | 2003 |
| Slovenia | NIB | 238 | Pear | 2003 |
| Slovenia | NIB | 239 | Apple | 2003 |
| Slovenia | NIB | 241 | Pear | 2003 |
| Slovenia | NIB | 242 | Apple | 2003 |
| Slovenia | NIB | 244 | Pear | 2003 |
| Slovenia | NIB | 245 | *Cydonia* sp. | 2003 |
| Slovenia | NIB | 246 | Apple | 2003 |
| Slovenia | NIB | 251 | Pear | 2003 |
| Slovenia | NIB | 252 | Pear | 2003 |
| Slovenia | NIB | 253 | *Crataegus* sp. | 2003 |
| Slovenia | NIB | 254 | Pear | 2003 |
| Slovenia | NIB | 257 | Apple | 2003 |
| Slovenia | NIB | 258 | Apple | 2003 |
| Slovenia | NIB | 259 | Pear | 2003 |
| Slovenia | NIB | 261 | Apple | 2003 |
| Slovenia | NIB | 262 | *Cotoneaster* sp. | 2003 |
| Slovenia | NIB | 264 | Pear | 2003 |
| Slovenia | NIB | 265 | Apple | 2003 |
| Slovenia | NIB | 271 | Pear | 2003 |
| Slovenia | NIB | 273 | Apple | 2003 |
| Slovenia | NIB | 274 | Pear | 2003 |
| Slovenia | NIB | 275 | Apple | 2003 |
| Slovenia | NIB | 278 | Pear | 2003 |
| Slovenia | NIB | 283 | Pear | 2003 |
| Slovenia | NIB | 284 | Apple | 2003 |
| Slovenia | NIB | 285 | Pear | 2003 |
| Slovenia | NIB | 286 | Pear | 2003 |
| Slovenia | NIB | 290 | Apple | 2003 |
| Slovenia | NIB | 291 | Apple | 2003 |
| Slovenia | NIB | 293 | Apple | 2003 |
| Slovenia | NIB | 298 | Apple | 2003 |
| Slovenia | NIB | 300 | Apple | 2003 |
| Slovenia | NIB | 301 | Apple | 2003 |
| Slovenia | NIB | 302 | *Cotoneaster* sp. | 2003 |
| Slovenia | NIB | 303 | Apple | 2003 |
| Slovenia | NIB | 311 | Pear | 2003 |
| Slovenia | NIB | 312 | Apple | 2003 |
| Slovenia | NIB | 313 | Apple | 2003 |
| Slovenia | NIB | 314 | Pear | 2003 |
| Slovenia | NIB | 315 | *Cotoneaster* sp. | 2003 |
| Slovenia | NIB | 316 | Pear | 2003 |
| Slovenia | NIB | 317 | Pear | 2003 |
| Slovenia | NIB | 318 | *Cydonia* sp. | 2003 |
| Slovenia | NIB | 321 | *Cydonia* sp. | 2003 |
| Slovenia | NIB | 323 | *Cotoneaster* sp. | 2003 |
| Slovenia | NIB | 324 | *Cydonia* sp. | 2003 |
| Slovenia | NIB | 325 | Apple | 2003 |
| Slovenia | NIB | 331 | *Cydonia* sp. | 2003 |
| Slovenia | NIB | 332 | *Cotoneaster* sp. | 2003 |
| Slovenia | NIB | 333 | *Cydonia* sp. | 2003 |
| Slovenia | NIB | 343 | *Cydonia* sp. | 2003 |
| Slovenia | NIB | 345 | Pear | 2003 |
| Slovenia | NIB | 346 | Pear | 2003 |
| Slovenia | NIB | 348 | *Cydonia* sp. | 2003 |
| Slovenia | NIB | 349 | Pear | 2003 |
| Slovenia | NIB | 350 | Apple | 2003 |
| Slovenia | NIB | 352 | Pear | 2003 |
| Slovenia | NIB | 355 | Apple | 2003 |
| Slovenia | NIB | 356 | Apple | 2003 |
| Slovenia | NIB | 357 | *Cydonia* sp. | 2003 |
| Slovenia | NIB | 359 | *Cydonia* sp. | 2003 |
| Slovenia | NIB | 360 | *Cydonia* sp. | 2003 |
| Slovenia | NIB | 363 | *Cydonia* sp. | 2003 |
| Slovenia | NIB | 364 | *Pyracantha* sp. | 2003 |
| Slovenia | NIB | 367 | *Cotoneaster* sp. | 2003 |
| Slovenia | NIB | 368 | *Cydonia* sp. | 2003 |
| Slovenia | NIB | 376 | Pear | 2003 |
| Slovenia | NIB | 378 | *Cotoneaster* sp. | 2003 |
| Slovenia | NIB | 379 | *Cotoneaster* sp. | 2003 |
| Slovenia | NIB | 380 | *Cydonia* sp. | 2003 |
| Slovenia | NIB | 382 | *Cydonia* sp. | 2003 |
| Slovenia | NIB | 383 | Pear | 2003 |
| Slovenia | NIB | 384 | Pear | 2003 |
| Slovenia | NIB | 385 | Apple | 2003 |
| Slovenia | NIB | 386 | *Cotoneaster* sp. | 2003 |
| Slovenia | NIB | 387 | Pear | 2003 |
| Slovenia | NIB | 388 | *Cotoneaster* sp. | 2003 |
| Slovenia | NIB | 389 | *Cydonia* sp. | 2003 |
| Slovenia | NIB | 391 | *Cydonia* sp. | 2003 |
| Slovenia | NIB | 392 | *Cydonia* sp. | 2003 |
| Slovenia | NIB | 394 | Apple | 2003 |
| Slovenia | NIB | 398 | *Cydonia* sp. | 2003 |
| Slovenia | NIB | 400 | *Cydonia* sp. | 2003 |
| Slovenia | NIB | 407 | *Cotoneaster* sp. | 2003 |
| Slovenia | NIB | 411 | Apple | 2003 |
| Slovenia | NIB | 412 | *Cotoneaster* sp. | 2003 |
| Slovenia | NIB | 413 | *Cotoneaster* sp. | 2003 |
| Slovenia | NIB | 424 | Pear | 2003 |
| Slovenia | NIB | 426 | Apple | 2003 |
| Slovenia | NIB | 430 | Pear | 2003 |
| Slovenia | NIB | 457 | *Cydonia* sp. | 2003 |
| Slovenia | NIB | 458 | *Cydonia* sp. | 2003 |
| Slovenia | NIB | 612 | *Cotoneaster* sp. | 2004 |
| Slovenia | NIB | 613 | *Cotoneaster* sp. | 2004 |
| Slovenia | NIB | 637 | *Cotoneaster* sp. | 2004 |
| Slovenia | NIB | 640 | Pear | 2004 |
| Slovenia | NIB | 641 | Pear | 2004 |
| Slovenia | NIB | 642 | Pear | 2004 |
| Slovenia | NIB | 649 | Pear | 2004 |
| Slovenia | NIB | 652 | *Cotoneaster* sp. | 2004 |
| Slovenia | NIB | 653 | Pear | 2004 |
| Slovenia | NIB | 736 | Pear | 2005 |
| Slovenia | NIB | 737 | Pear | 2005 |
| Slovenia | NIB | 739 | Pear | 2005 |
| Slovenia | NIB | 740 | *Cotoneaster* sp. | 2005 |
| Slovenia | NIB | 754 | *Cotoneaster* sp. | 2005 |
| Slovenia | NIB | 763 | *Cotoneaster* sp. | 2005 |
| Slovenia | NIB | 811 | Apple | 2006 |
| Slovenia | NIB | 883 | Apple | 2007 |
| Slovenia | NIB | 884 | Apple | 2007 |
| Slovenia | NIB | 885 | Apple | 2007 |
| Slovenia | NIB | 886 | Apple | 2007 |
| Slovenia | NIB | 887 | *Cydonia* sp. | 2007 |
| Slovenia | NIB | 888 | *Cydonia* sp. | 2007 |
| Slovenia | NIB | 889 | *Cydonia* sp. | 2007 |
| Slovenia | NIB | 890 | *Cydonia* sp. | 2007 |
| Slovenia | NIB | 893 | Apple | 2007 |
| Slovenia | NIB | 895 | *Cydonia* sp. | 2007 |
| Slovenia | NIB | 896 | Pear | 2007 |
| Slovenia | NIB | 898 | Pear | 2007 |
| Slovenia | NIB | 899 | Pear | 2007 |
| Slovenia | NIB | 900 | Pear | 2007 |
| Slovenia | NIB | 904 | Apple | 2007 |
| Slovenia | NIB | 905 | Pear | 2007 |
| Slovenia | NIB | 907 | Apple | 2007 |
| Slovenia | NIB | 914 | *Crataegus* sp. | 2007 |
| Slovenia | NIB | 915 | Pear | 2007 |
| Slovenia | NIB | 916 | Apple | 2007 |
| Slovenia | NIB | 921 | *Crataegus* sp. | 2007 |
| Slovenia | NIB | 922 | *Cydonia* sp. | 2007 |
| Slovenia | NIB | 923 | Pear | 2007 |
| Slovenia | NIB | 924 | Pear | 2007 |
| Slovenia | NIB | 928 | Apple | 2007 |
| Slovenia | NIB | 929 | Apple | 2007 |
| Slovenia | NIB | 930 | Pear | 2007 |
| Slovenia | NIB | 931 | Pear | 2007 |
| Slovenia | NIB | 932 | Pear | 2007 |
| Slovenia | NIB | 933 | Apple | 2007 |
| Slovenia | NIB | 934 | Apple | 2007 |
| Slovenia | NIB | 953 | Apple | 2007 |
| Slovenia | NIB | 955 | Apple | 2007 |
| Slovenia | NIB | 956 | Apple | 2007 |
| Slovenia | NIB | 957 | Apple | 2007 |
| Slovenia | NIB | 961 | *Cydonia* sp. | 2007 |
| Slovenia | NIB | 962 | Pear | 2007 |
| Slovenia | NIB | 963 | *Cotoneaster* sp. | 2007 |
| Slovenia | NIB | 964 | Pear | 2007 |
| Slovenia | NIB | 965 | Pear | 2007 |
| Slovenia | NIB | 967 | Apple | 2007 |
| Slovenia | NIB | 968 | Apple | 2007 |
| Slovenia | NIB | 970 | Pear | 2007 |
| Slovenia | NIB | 971 | Apple | 2007 |
| Slovenia | NIB | 973 | Apple | 2007 |
| Slovenia | NIB | 974 | Pear | 2007 |
| Slovenia | NIB | 975 | Apple | 2007 |
| Slovenia | NIB | 976 | Apple | 2007 |
| Slovenia | NIB | 977 | Apple | 2007 |
| Slovenia | NIB | 978 | Apple | 2007 |
| Slovenia | NIB | 979 | Pear | 2007 |
| Slovenia | NIB | 982 | Pear | 2007 |
| Slovenia | NIB | 983 | Pear | 2007 |
| Slovenia | NIB | 984 | *Cydonia* sp. | 2007 |
| Slovenia | NIB | 985 | *Cotoneaster* sp. | 2007 |
| Slovenia | NIB | 986 | Apple | 2007 |
| Slovenia | NIB | 987 | Pear | 2007 |
| Slovenia | NIB | 988 | Pear | 2007 |
| Slovenia | NIB | 989 | Apple | 2007 |
| Slovenia | NIB | 990 | Pear | 2007 |
| Slovenia | NIB | 991 | Pear | 2007 |
| Slovenia | NIB | 1000 | Pear | 2007 |
| Slovenia | NIB | 1001 | Pear | 2007 |
| Slovenia | NIB | 1002 | Apple | 2007 |
| Slovenia | NIB | 1003 | Apple | 2007 |
| Slovenia | NIB | 1004 | Apple | 2007 |
| Slovenia | NIB | 1006 | Pear | 2007 |
| Slovenia | NIB | 1007 | Pear | 2007 |
| Slovenia | NIB | 1010 | *Cydonia* sp. | 2007 |
| Slovenia | NIB | 1012 | Pear | 2007 |
| Slovenia | NIB | 1013 | Pear | 2007 |
| Slovenia | NIB | 1014 | Pear | 2007 |
| Slovenia | NIB | 1015 | Pear | 2007 |
| Slovenia | NIB | 1016 | Pear | 2007 |
| Slovenia | NIB | 1017 | Pear | 2007 |
| Slovenia | NIB | 1018 | Pear | 2007 |
| Slovenia | NIB | 1019 | Pear | 2007 |
| Slovenia | NIB | 1020 | Pear | 2007 |
| Slovenia | NIB | 1021 | Pear | 2007 |
| Slovenia | NIB | 1022 | Pear | 2007 |
| Slovenia | NIB | 1023 | *Cydonia* sp. | 2007 |
| Slovenia | NIB | 1025 | Pear | 2007 |
| Slovenia | NIB | 1026 | Pear | 2007 |
| Slovenia | NIB | 1027 | Apple | 2007 |
| Slovenia | NIB | 1030 | Pear | 2007 |
| Slovenia | NIB | 1031 | Pear | 2007 |
| Slovenia | NIB | 1032 | Pear | 2007 |
| Slovenia | NIB | 1034 | Pear | 2007 |
| Slovenia | NIB | 1035 | Pear | 2007 |
| Slovenia | NIB | 1036 | *Cydonia* sp. | 2007 |
| Slovenia | NIB | 1038 | Pear | 2007 |
| Slovenia | NIB | 1039 | Pear | 2007 |
| Slovenia | NIB | 1040 | *Cydonia* sp. | 2007 |
| Slovenia | NIB | 1042 | Pear | 2007 |
| Slovenia | NIB | 1043 | Pear | 2007 |
| Slovenia | NIB | 1044 | *Cotoneaster* sp. | 2007 |
| Slovenia | NIB | 1045 | Pear | 2007 |
| Slovenia | NIB | 1047 | Pear | 2007 |
| Slovenia | NIB | 1054 | *Cydonia* sp. | 2007 |
| Slovenia | NIB | 1057 | Pear | 2007 |
| Slovenia | NIB | 1058 | Pear | 2007 |
| Slovenia | NIB | 1060 | Pear | 2007 |
| Slovenia | NIB | 1061 | Apple | 2007 |
| Slovenia | NIB | 1062 | Pear | 2007 |
| Slovenia | NIB | 1063 | Pear | 2007 |
| Slovenia | NIB | 1064 | Pear | 2007 |
| Slovenia | NIB | 1065 | Pear | 2007 |
| Slovenia | NIB | 1069 | Pear | 2007 |
| Slovenia | NIB | 1070 | Pear | 2007 |
| Slovenia | NIB | 1071 | Pear | 2007 |
| Slovenia | NIB | 1072 | Pear | 2007 |
| Slovenia | NIB | 1076 | Pear | 2007 |
| Slovenia | NIB | 1077 | Pear | 2007 |
| Slovenia | NIB | 1083 | Pear | 2007 |
| Slovenia | NIB | 1084 | Pear | 2007 |
| Slovenia | NIB | 1085 | Pear | 2007 |
| Slovenia | NIB | 1088 | Pear | 2007 |
| Slovenia | NIB | 1092 | Pear | 2007 |
| Slovenia | NIB | 1094 | Pear | 2007 |
| Slovenia | NIB | 1095 | Pear | 2007 |
| Slovenia | NIB | 1096 | Pear | 2007 |
| Slovenia | NIB | 1097 | Pear | 2007 |
| Slovenia | NIB | 1098 | Pear | 2007 |
| Slovenia | NIB | 1099 | Pear | 2007 |
| Slovenia | NIB | 1101 | *Cydonia* sp. | 2007 |
| Slovenia | NIB | 1112 | *Photinia* sp. | 2007 |
| Slovenia | NIB | 1113 | Pear | 2007 |
| Slovenia | NIB | 1114 | Pear | 2007 |
| Slovenia | NIB | 1115 | Pear | 2007 |
| Slovenia | NIB | 1116 | Pear | 2007 |
| Slovenia | NIB | 1117 | Apple | 2007 |
| Slovenia | NIB | 1118 | *Cydonia* sp. | 2007 |
| Slovenia | NIB | 1119 | Apple | 2007 |
| Slovenia | NIB | 1120 | Apple | 2007 |
| Slovenia | NIB | 1121 | Apple | 2007 |
| Slovenia | NIB | 1122 | Apple | 2007 |
| Slovenia | NIB | 1127 | *Cydonia* sp. | 2007 |
| Slovenia | NIB | 1128 | *Cotoneaster* sp. | 2007 |
| Slovenia | NIB | 1129 | Pear | 2007 |
| Slovenia | NIB | 1136 | Pear | 2007 |
| Slovenia | NIB | 1137 | Pear | 2007 |
| Slovenia | NIB | 1138 | Pear | 2007 |
| Slovenia | NIB | 1139 | Pear | 2007 |
| Slovenia | NIB | 1201 | Apple | 2008 |
| Slovenia | NIB | 1203 | Pear | 2008 |
| Slovenia | NIB | 1204 | Apple | 2008 |
| Slovenia | NIB | 1205 | Apple | 2008 |
| Slovenia | NIB | 1206 | Pear | 2008 |
| Slovenia | NIB | 1207 | Apple | 2008 |
| Slovenia | NIB | 1212 | Apple | 2008 |
| Slovenia | NIB | 1213 | Apple | 2008 |
| Slovenia | NIB | 1215 | Pear | 2008 |
| Slovenia | NIB | 1218 | Pear | 2008 |
| Slovenia | NIB | 1219 | Pear | 2008 |
| Slovenia | NIB | 1220 | Pear | 2008 |
| Slovenia | NIB | 1221 | Apple | 2008 |
| Slovenia | NIB | 1222 | Pear | 2008 |
| Slovenia | NIB | 1225 | Pear | 2008 |
| Slovenia | NIB | 1226 | *Cydonia* sp. | 2008 |
| Slovenia | NIB | 1227 | Apple | 2008 |
| Slovenia | NIB | 1228 | Apple | 2008 |
| Slovenia | NIB | 1229 | *Cydonia* sp. | 2008 |
| Slovenia | NIB | 1230 | Pear | 2008 |
| Slovenia | NIB | 1261 | Apple | 2008 |
| Slovenia | NIB | 1262 | Apple | 2008 |
| Slovenia | NIB | 1264 | Pear | 2008 |
| Slovenia | NIB | 1265 | *Cydonia* sp. | 2008 |
| Slovenia | NIB | 1276 | Pear | 2008 |
| Slovenia | NIB | 1277 | Pear | 2008 |
| Slovenia | NIB | 1278 | Apple | 2008 |
| Slovenia | NIB | 1279 | Pear | 2008 |
| Slovenia | NIB | 1321 | Pear | 2009 |
| Slovenia | NIB | 1327 | Pear | 2009 |
| Slovenia | NIB | 1340 | Pear | 2009 |
| Slovenia | NIB | 1345 | Pear | 2009 |
| Spain | IVIA | IVIA1554-1 | *Crataegus* sp. | 1996 |
| Spain | IVIA | IVIA1554-2 | *Crataegus* sp. | 1996 |
| Spain | IVIA | IVIA 1578-3 | *Crataegus* sp. | 1996 |
| Spain | IVIA | IVIA 1596 | *Pear* | 1996 |
| Spain | IVIA | IVIA 1603 | *Cotoneaster* sp. | 1996 |
| Spain | IVIA | IVIA 1614-1 | Pyracantha sp. | 1996 |
| Spain | IVIA | IVIA 1614-2a | *Crataegus* sp. | 1996 |
| Spain | IVIA | IVIA 1892-1 | Pear | 1998 |
| Spain | IVIA | IVIA 1898-11 | Apple | 1998 |
| Spain | IVIA | IVIA 1898-18 | Apple | 1998 |
| Spain | IVIA | IVIA 1899-19 | Pear | 1998 |
| Spain | IVIA | IVIA 1985 | *Pyracantha* sp. | 1998 |
| Spain | IVIA | IVIA 2397 | Pear | 2000 |
| Spain | IVIA | IVIA 3169 | Pear | 2006 |
| Spain | IVIA | IVIA 3171 | *Crataegus* sp. | 2006 |
| Spain | IVIA | IVIA 3172 | Pear | 2006 |
| Spain | IVIA | IVIA 3173 | Apple | 2006 |
| Spain | IVIA | IVIA 3174 | *Crataegus* sp. | 2006 |
| Spain | IVIA | IVIA 3199-4 | *Crataegus* sp. | 2006 |
| Spain | UPN | UPN 538 | Pear | 1998 |
| Switzerland | ACW | Rac 3075 | na | na |
| Switzerland | ACW | 35697 | Pear | 2003 |
| Switzerland | ACW | 37678 | Pear | 2003 |
| Switzerland | ACW | 37809 | Pear | 2003 |
| Switzerland | ACW | 38262 | *Crataegus* sp. | 2003 |
| Switzerland | ACW | 38899 | *Cydonia* sp. | 2003 |
| Switzerland | ACW | 39005 | *Pyracantha* sp. | 2003 |
| Switzerland | ACW | 44081 | Apple | 2005 |
| Switzerland | ACW | 44274 | Pear | 2005 |
| Switzerland | ACW | 45072 | Pear | 2005 |
| Switzerland | ACW | 45534 | *Crataegus* sp. | 2005 |
| Switzerland | ACW | 56400 | Pear | 2007 |
| Switzerland | ACW | 56437 | Pear | 2007 |
| The Netherlands | CFBP | CFBP 3017 | *Crataegus* sp. | 1973 |
| The Netherlands | CFBP | CFBP 3021 | Pear | 1981 |
| UK | NCPPB | NCPPB 3299 | Pernettya | 1983 |
| UK | BBA | P-1570 | *Cotoneaster* sp. | na |

na: not available

ACW: Agroscope-Changins Wädenswil, Switzerland.

BBA: Federal Biological Institute for Agriculture and Forestry. Dossenheim, Germany.

BPIC: Benaki Phytopathological Institute Collection, Athens, Greece.

CFBP: Collection Française de Bactéries Phytopathogènes, INRA, Angers, France.

CRA-W : Centre Wallon de Recherches Agronomiques. Gembloux, Belgium.

DERM: Department of Environmental Resource Management, University College Dublin, Belfield, Ireland.

DPP: Department of Plant Protection collection, Institute of Horticulture, Pomology Divison, Skiernewice, Poland.

IVIA: Instituto Valenciano de Investigaciones Agrarias collection, Moncada, Spain.

LMG: Collection of the Laboratory of Microbiology. Rijksuniversiteit, Gent, Belgium.

LNPV: Laboratoire National de la Protection des Végétaux, Beaucouzé, France.

#### NCPPB: National Collection of Plant Pathogenic Bacteria, York, UK.

NIB: National Institute of Biology, Ljubljana, Slovenia.

OMP: Osservatorio per le Malattie delle Piante, Bologna, Italy.

#### SFR: Servizio Fitosanitario Regionale, Bologna, Italy.

SL: State Laboratory, Dublin, Ireland.

UPN: Universidad Publica de Navarra. Pamplona, Spain.
