# Supplementary material for: Erwinia amylovora Novel Plasmid pEI70: Complete Sequence, Biogeography, and Role in Aggressiveness in the Fire Blight Phytopathogen
Source: PLoS One. 2011 Dec 9;6(12):e28651. doi: 10.1371/journal.pone.0028651 (PMC3235134; doi:10.1371/journal.pone.0028651)
Supplement: Table S3 — Additional information available from E. amylovora strains negative for pEI70 analyzed in European countries. (PDF) [file pone.0028651.s003.pdf]

Table S3.

Country	Collection	Isolates negative for pEI70	Host	Year of isolation
Austria	AGES	MK 295/3	*Cotoneaster* sp.	1993
Austria	AGES	MK 483/98	*Cotoneaster* sp.	1998
Austria	AGES	MK 1082/00	Pear	2000
Austria	AGES	MK 1180/00	Apple	2000
Austria	AGES	MK 1186/00	*Sorbus* sp.	2000
Austria	AGES	MK 2447/01	*Sorbus* sp.	2001
Austria	AGES	MK 295/83 (4)	*Cotoneaster* sp.	1993
Austria	AGES	MK 295/83 (54)	*Cotoneaster* sp.	1993
Austria	AGES	MK 295/93 (127)	*Cotoneaster* sp.	1993
Austria	AGES	MK 2694/01	Apple	2001
Austria	AGES	MK 2677/01	Apple	2001
Austria	AGES	MK 273/98	Apple	1998
Austria	AGES	MK 296/93	*Cotoneaster* sp.	1993
Austria	AGES	MK 324/98	Apple	1998
Austria	AGES	MK 328/98	*Cotoneaster* sp.	1998
Austria	AGES	MK 482/98	*Cotoneaster* sp.	1998
Austria	AGES	MK 640/99	Apple	1999
Austria	AGES	MK 668/00	Apple	2000
Austria	AGES	MK674/94	Pear	1994
Austria	AGES	MK 881/98	na	1998
Austria	AGES	MK 955/99	Pear	1999
Austria	AGES	MK 1059/03	Pear	2003
Austria	AGES	MK 1057/03	Apple	2003
Austria	AGES	MK 2057/02	*Prunus* sp.	2002
Austria	AGES	MK 2614/01	Apple	2001
Belgium	LMG	LMG 1930	*Crataegus* sp.	2001
Belgium	CRA-W	EaP3	*Crataegus* sp.	1980
Belgium	CRA-W	EaA22	Pear	2001
Belgium	CRA-W	EaA23	*Crataegus* sp.	2001
Belgium	CRA-W	EaP45	Pear	2002
Bulgaria	CRA-W	KG 115.22a	*Cydonia* sp.	na
Bulgaria	CRA-W	KG 115.22b	*Cydonia* sp.	na
Bulgaria	BPIC	BPIC 1041	*Cydonia* sp.	1991
Czech Republic	BPIC	BPIC 1634	*Crataegus* sp.	1989
France	CFBP	CFBP1430	*Crataegus* sp.	1972
France	CFBP	CFBP 2314	Apple	1982
France	CFBP	CFBP 2597(1)	Apple	na
France	INRA	INRA 2543	Apple	1992
France	INRA	INRA 2546 (p)	Apple	1992
France	INRA	INRA 2550 (p)	Apple	1992
France	INRA	INRA 2552 (p)	Apple	1992
France	INRA	INRA 2553 (p)	na	na
France	INRA	INRA 2561 (p)	na	na

France	INRA	INRA 2576 (p)	Pear	na
France	INRA	INRA 2579 (p)	Apple	na
France	INRA	INRA 2580 (p)	Apple	na
France	INRA	INRA 2581	Apple	1992
France	INRA	INRA 2582	Apple	1992
France	INRA	INRA 2595	*Cotoneaster* sp.	na
France	BBA	KG 6-45	*Cydonia* sp.	na
France	BBA	KG 9-43	*Cotoneaster* sp.	na
France	BBA	KG 9-7	Pear	na
France	BBA	KG 9-75	*Cotoneaster* sp.	na
France	LNPV	LNPV 1099	*Pyracantha* sp.	na
France	LNPV	LNPV 1594	*Cotoneaster* sp.	1993
France	LNPV	LNPV 1601	Pear	1993
France	LNPV	LNPV 1613	*Pyracantha* sp.	1993
France	LNPV	LNPV 1626	Pear	1993
France	LNPV	LNPV 1709	*Cotoneaster* sp.	na
France	LNPV	LNPV 1710	*Cotoneaster* sp.	na
France	LNPV	LNPV 1775	*Cotoneaster* sp.	na
France	LNPV	LNPV 1778	*Cotoneaster* sp.	1993
France	LNPV	LNPV 1887	*Cotoneaster* sp.	na
France	LNPV	LNPV 1888	*Cotoneaster* sp.	na
France	LNPV	LNPV1890	*Cotoneaster* sp.	na
France	LNPV	LNPV 1892	*Cotoneaster* sp.	na
France	LNPV	LNPV 1893	*Cotoneaster* sp.	na
France	LNPV	LNPV 1894	*Cotoneaster* sp.	na
France	LNPV	LNPV 1899	*Cotoneaster* sp.	na
France	LNPV	LNPV 2018	*Cotoneaster* sp.	na
France	LNPV	LNPV 2026	*Cotoneaster* sp.	na
France	LNPV	LNPV 2030	*Pyracantha* sp.	na
France	LNPV	LNPV 1257-B	*Photinia* sp.	na
France	LNPV	LNPV 1096	*Cotoneaster* sp.	na
Germany	BBA	Ea 115	Apple	1989
Germany	CFBP	CFBP 3053	Apple	1981
Germany	CFBP	CFBP 3054	Pear	1974
Germany	CFBP	CFBP 3057	*Cotoneaster* sp.	1974
Germany	BBA	KG 237	Apple	na
Germany	BBA	KG 250	Apple	na
Germany	BBA	KG 282	Pear	na
Germany	BBA	KG 286	Pear	na
Germany	BBA	KG 1/79	*Cotoneaster* sp.	na
Germany	BBA	KG FR-3/98	*Cotoneaster* sp.	na
Germany	AGES	MK DL 1	Apple	1993
Germany	AGES	MK 7/74	*Cotoneaster* sp.	1974
Greece	BPIC	BPIC 909	Pear	1985
Greece	BPIC	BPIC 917	*Cydonia* sp.	1985
Greece	BPIC	BPIC 913	Pear	1985
Greece	BPIC	BPIC 1624	Pear	1990
Greece	BPIC	BPIC 1614	Pear	1990

Greece	BPIC	BPIC 943	Pear	1984
Greece	BPIC	BPIC 932	Apple	1986
Greece	BPIC	BPIC 928	Pear	1986
Greece	BPIC	BPIC 939	Pear	1986
Greece	BPIC	BPIC 847	Pear	1984
Greece	BPIC	BPIC 1056	*Cydonia* sp.	1987
Greece	CFBP	CFBP 3063	Pear	1985
Greece	CFBP	CFBP 3064	Pear	1985
Greece	CFBP	CFBP 3065	Pear	1986
Greece	CFBP	CFBP 3093	Pear	1986
Hungary	AGES	MK 1	Apple	1996
Hungary	AGES	MK 10	Pear	1997
Hungary	AGES	MK 15	*Cotoneaster* sp.	1997
Hungary	AGES	MK 16	*Cotoneaster* sp.	1997
Hungary	AGES	MK 17	*Cydonia* sp.	1997
Hungary	AGES	MK 22	*Crataegus* sp.	1998
Hungary	AGES	MK 23	Pear	1998
Hungary	AGES	MK 26	Pear	1998
Hungary	AGES	MK 28	*Crataegus* sp.	1998
Hungary	AGES	MK 31	*Pyracantha* sp.	1998
Hungary	AGES	MK 1	Apple	1996
Ireland	SL	AE 91	Pear	na
Ireland	SL	E-31	*Cotoneaster* sp.	1996
Italy	OMP	OMP-BO 787-1	*Crataegus* sp.	1992
Italy	OMP	OMP-BO 1077-7	Pear	1994
Poland	DPP	123	*Crataegus* sp.	2000
Poland	DPP	462	Apple	2002
Poland	DPP	464	Pear	2002
Poland	DPP	518	Pear	2002
Poland	DPP	538	Apple	2002
Poland	DPP	576	*Crataegus* sp.	2002
Poland	DPP	604	*Crataegus* sp.	1994
Poland	DPP	607	Apple	1994
Poland	DPP	608	Apple	1994
Poland	DPP	611	Apple	1995
Poland	DPP	612	Pear	1995
Poland	DPP	613	*Crataegus* sp.	1995
Poland	DPP	614	Apple	1995
Poland	DPP	615	*Crataegus* sp.	1995
Poland	DPP	616	Apple	1995
Poland	DPP	617	*Crataegus* sp.	1995
Poland	DPP	618	Pear	1995
Poland	DPP	619	Pear	1995
Poland	DPP	620	*Cotoneaster* sp.	1995
Poland	DPP	621	Pear	1995
Poland	DPP	622	*Cydonia* sp.	1995
Poland	DPP	623	*Crataegus* sp.	1995
Poland	DPP	624	Pear	1995

Poland	DPP	625	Pear	1995
Poland	DPP	627	Apple	1985
Poland	DPP	629	Apple	1985
Poland	DPP	632	Pear	1985
Poland	DPP	633	Pear	1985
Poland	DPP	634	*Crataegus* sp.	1985
Poland	DPP	635	*Crataegus* sp.	1985
Poland	DPP	636	*Crataegus* sp.	1985
Poland	DPP	637	Pear	1985
Poland	DPP	645	*Crataegus* sp.	1985
Poland	DPP	646	*Crataegus* sp.	1985
Poland	DPP	648	*Crataegus* sp.	1983
Poland	DPP	650	*Crataegus* sp.	1983
Poland	DPP	651	*Crataegus* sp.	1983
Poland	DPP	659	Apple	1986
Poland	DPP	660	Pear	1993
Poland	DPP	661	*Sorbus* sp.	1993
Poland	DPP	663	Apple	1993
Poland	DPP	664	Apple	1993
Poland	DPP	684	*Crataegus* sp.	1997
Poland	DPP	691	Apple	1998
Poland	DPP	692	*Sorbus* sp.	1998
Poland	DPP	694	Apple	2000
Poland	DPP	698	Apple	na
Poland	DPP	39/675	*Crataegus* sp.	2000
Poland	DPP	133/95	Quince	1995
Poland	DPP	16/96	Apple	1996
Poland	DPP	21/96	Pear	1996
Poland	DPP	2112/00	Pear	2000
Poland	DPP	2119/00	*Crataegus* sp.	2000
Poland	DPP	244/96	*Cotoneaster* sp.	1996
Poland	DPP	367/96	*Pyracantha* sp.	1996
Poland	DPP	74/95	*Sorbus* sp.	1995
Poland	DPP	96/96	*Sorbus* sp.	1996
Poland	DPP	E 26	Apple	2001
Poland	DPP	E1/I	Apple	1999
Poland	DPP	E1/II	Apple	1999
Poland	DPP	E10	Apple	2000
Poland	DPP	E11	Apple	2000
Poland	DPP	E12	Apple	2000
Poland	DPP	E13	Apple	2000
Poland	DPP	E14	Pear	2000
Poland	DPP	E15	Pear	2000
Poland	DPP	E16	Apple	2000
Poland	DPP	E17	Apple	2000
Poland	DPP	E18	Apple	2000
Poland	DPP	E19	Apple	2000
Poland	DPP	E2/I	Apple	2000

Poland	DPP	E2/II	Apple	2000
Poland	DPP	E20	Apple	2000
Poland	DPP	E21	Apple	2000
Poland	DPP	E22	na	2000
Poland	DPP	E23	Apple	2001
Poland	DPP	E24	Apple	2002
Poland	DPP	E25	Pear	2002
Poland	DPP	E27	Apple	2002
Poland	DPP	E28	Apple	2002
Poland	DPP	E29	Apple	2002
Poland	DPP	E3	Apple	2000
Poland	DPP	E4	Pear	2000
Poland	DPP	E5	Pear	2000
Poland	DPP	E6	Pear	2000
Poland	DPP	E7	Pear	2000
Poland	DPP	E8	Pear	2000
Poland	DPP	E9	Apple	2000
Poland	DPP	Pr 591	*Crataegus* sp.	2000
Poland	DPP	Pr 82	*Crataegus* sp.	2000
Poland	DPP	TL 81	Apple	2000
Poland	DPP	TL 85	Apple	2000
Poland	DPP	TL 86	Apple	2000
Poland	DPP	TN44	*Crataegus* sp.	2000
Poland	DPP	604a	Apple	2001
Poland	DPP	608a	Apple	2002
Poland	DPP	609a	Pear	2003
Poland	DPP	610a	Apple	2003
Poland	DPP	611a	Pear	2003
Poland	DPP	613a	Pear	2004
Poland	DPP	614a	Pear	2004
Poland	DPP	615a	Pear	2006
Poland	DPP	616a	Pear	2006
Poland	DPP	617a	Pear	2005
Poland	DPP	618a	Apple	2007
Poland	DPP	620a	Apple	2007
Poland	DPP	621a	Apple	2007
Poland	DPP	622a	Apple	2007
Poland	DPP	623a	Apple	2007
Poland	DPP	624a	Pear	2007
Poland	DPP	626a	Apple	2007
Poland	DPP	627a	Apple	2007
Poland	DPP	629a	Apple	2007
Serbia	ARI	BC-3	Apple	2003
Serbia	ARI	CGJ-2	Apple	2003
Slovenia	NIB	174	Apple	2003
Slovenia	NIB	179	*Cotoneaster* sp.	2003
Slovenia	NIB	186	Apple	2003
Slovenia	NIB	187	Apple	2003

Slovenia	NIB	188	Apple	2003
Slovenia	NIB	194	Apple	2003
Slovenia	NIB	196	Pear	2003
Slovenia	NIB	197	Apple	2003
Slovenia	NIB	198	Apple	2003
Slovenia	NIB	200	Pear	2003
Slovenia	NIB	201	Apple	2003
Slovenia	NIB	209	Apple	2003
Slovenia	NIB	214	Apple	2003
Slovenia	NIB	227	Apple	2003
Slovenia	NIB	232	Apple	2003
Slovenia	NIB	233	Pear	2003
Slovenia	NIB	234	Apple	2003
Slovenia	NIB	240	Apple	2003
Slovenia	NIB	243	Apple	2003
Slovenia	NIB	256	Apple	2003
Slovenia	NIB	260	Pear	2003
Slovenia	NIB	263	Apple	2003
Slovenia	NIB	266	Apple	2003
Slovenia	NIB	267	Apple	2003
Slovenia	NIB	268	Apple	2003
Slovenia	NIB	269	Apple	2003
Slovenia	NIB	270	Apple	2003
Slovenia	NIB	276	Apple	2003
Slovenia	NIB	277	Apple	2003
Slovenia	NIB	279	Pear	2003
Slovenia	NIB	280	Apple	2003
Slovenia	NIB	281	Apple	2003
Slovenia	NIB	282	Apple	2003
Slovenia	NIB	292	Apple	2003
Slovenia	NIB	294	*Chaenomeles* sp.	2003
Slovenia	NIB	295	Apple	2003
Slovenia	NIB	299	Apple	2003
Slovenia	NIB	319	Apple	2003
Slovenia	NIB	320	Apple	2003
Slovenia	NIB	327	*Cydonia* sp.	2003
Slovenia	NIB	329	Apple	2003
Slovenia	NIB	334	Apple	2003
Slovenia	NIB	335	Apple	2003
Slovenia	NIB	336	Apple	2003
Slovenia	NIB	337	Apple	2003
Slovenia	NIB	338	Pear	2003
Slovenia	NIB	339	Apple	2003
Slovenia	NIB	340	Apple	2003
Slovenia	NIB	341	Pear	2003
Slovenia	NIB	342	Pear	2003
Slovenia	NIB	350	Apple	2003
Slovenia	NIB	353	*Cydonia* sp.	2003

Slovenia	NIB	354	Apple	2003
Slovenia	NIB	355	Apple	2003
Slovenia	NIB	356	Apple	2003
Slovenia	NIB	357	*Cydonia* sp.	2003
Slovenia	NIB	359	*Cydonia* sp.	2003
Slovenia	NIB	360	*Cydonia* sp.	2003
Slovenia	NIB	363	*Cydonia* sp.	2003
Slovenia	NIB	365	Apple	2003
Slovenia	NIB	366	Apple	2003
Slovenia	NIB	370	Apple	2003
Slovenia	NIB	371	*Cydonia* sp.	2003
Slovenia	NIB	374	Apple	2003
Slovenia	NIB	375	Apple	2003
Slovenia	NIB	401	*Cotoneaster* sp.	2003
Slovenia	NIB	402	*Cotoneaster* sp.	2003
Slovenia	NIB	403	*Cotoneaster* sp.	2003
Slovenia	NIB	404	Apple	2003
Slovenia	NIB	405	Apple	2003
Slovenia	NIB	414	*Cotoneaster* sp.	2003
Slovenia	NIB	415	*Cotoneaster* sp.	2003
Slovenia	NIB	416	*Cotoneaster* sp.	2003
Slovenia	NIB	417	*Cotoneaster* sp.	2003
Slovenia	NIB	425	Apple	2003
Slovenia	NIB	429	Pear	2003
Slovenia	NIB	609	Apple	2004
Slovenia	NIB	610	Apple	2004
Slovenia	NIB	611	Apple	2004
Slovenia	NIB	614	Apple	2004
Slovenia	NIB	615	Apple	2004
Slovenia	NIB	616	Apple	2004
Slovenia	NIB	617	Pear	2004
Slovenia	NIB	618	*Cotoneaster* sp.	2004
Slovenia	NIB	633	Apple	2004
Slovenia	NIB	634	Apple	2004
Slovenia	NIB	636	*Cotoneaster* sp.	2004
Slovenia	NIB	638	Apple	2004
Slovenia	NIB	639	na	2004
Slovenia	NIB	643	na	2004
Slovenia	NIB	647	Apple	2004
Slovenia	NIB	648	Apple	2004
Slovenia	NIB	651	Apple	2004
Slovenia	NIB	654	Pear	2004
Slovenia	NIB	655	Pear	2004
Slovenia	NIB	656	Pear	2004
Slovenia	NIB	687	*Cotoneaster* sp.	2004
Slovenia	NIB	731	Apple	2005
Slovenia	NIB	733	Apple	2005
Slovenia	NIB	734	Apple	2005

Slovenia	NIB	735	Pear	2005
Slovenia	NIB	738	Pear	2005
Slovenia	NIB	743	Pear	2005
Slovenia	NIB	747	Pear	2005
Slovenia	NIB	812	Apple	2006
Slovenia	NIB	813	Apple	2006
Slovenia	NIB	814	Apple	2006
Slovenia	NIB	815	Pear	2006
Slovenia	NIB	816	Pear	2006
Slovenia	NIB	882	Apple	2007
Slovenia	NIB	891	Pear	2007
Slovenia	NIB	892	Pear	2007
Slovenia	NIB	894	na	2007
Slovenia	NIB	897	Apple	2007
Slovenia	NIB	899	Pear	2007
Slovenia	NIB	901	Apple	2007
Slovenia	NIB	902	Pear	2007
Slovenia	NIB	903	Apple	2007
Slovenia	NIB	906	Apple	2007
Slovenia	NIB	908	Apple	2007
Slovenia	NIB	909	Apple	2007
Slovenia	NIB	910	Apple	2007
Slovenia	NIB	911	Apple	2007
Slovenia	NIB	912	Pear	2007
Slovenia	NIB	913	Pear	2007
Slovenia	NIB	917	Apple	2007
Slovenia	NIB	918	Apple	2007
Slovenia	NIB	919	Apple	2007
Slovenia	NIB	920	Apple	2007
Slovenia	NIB	925	Apple	2007
Slovenia	NIB	926	Apple	2007
Slovenia	NIB	927	Apple	2007
Slovenia	NIB	935	Apple	2007
Slovenia	NIB	954	Pear	2007
Slovenia	NIB	958	Apple	2007
Slovenia	NIB	959	Apple	2007
Slovenia	NIB	960	Apple	2007
Slovenia	NIB	966	Pear	2007
Slovenia	NIB	969	Pear	2007
Slovenia	NIB	972	Apple	2007
Slovenia	NIB	980	Apple	2007
Slovenia	NIB	981	Apple	2007
Slovenia	NIB	992	Pear	2007
Slovenia	NIB	993	Apple	2007
Slovenia	NIB	994	*Cydonia* sp.	2007
Slovenia	NIB	995	*Crataegeus* sp.	2007
Slovenia	NIB	996	Apple	2007
Slovenia	NIB	997	Apple	2007

Slovenia	NIB	998	Pear	2007
Slovenia	NIB	999	Apple	2007
Slovenia	NIB	1005	Apple	2007
Slovenia	NIB	1008	Pear	2007
Slovenia	NIB	1009	Apple	2007
Slovenia	NIB	1028	Apple	2007
Slovenia	NIB	1029	Pear	2007
Slovenia	NIB	1033	*Crataegus* sp.	2007
Slovenia	NIB	1037	Pear	2007
Slovenia	NIB	1046	Pear	2007
Slovenia	NIB	1048	Apple	2007
Slovenia	NIB	1049	Pear	2007
Slovenia	NIB	1050	Pear	2007
Slovenia	NIB	1051	Pear	2007
Slovenia	NIB	1053	Apple	2007
Slovenia	NIB	1055	Pear	2007
Slovenia	NIB	1059	Pear	2007
Slovenia	NIB	1066	Pear	2007
Slovenia	NIB	1067	Pear	2007
Slovenia	NIB	1075	*Cydonia* sp.	2007
Slovenia	NIB	1078	Pear	2007
Slovenia	NIB	1081	Pear	2007
Slovenia	NIB	1082	Pear	2007
Slovenia	NIB	1090	Pear	2007
Slovenia	NIB	1093	Pear	2007
Slovenia	NIB	1100	*Cydonia* sp.	2007
Slovenia	NIB	1123	Apple	2007
Slovenia	NIB	1124	Pear	2007
Slovenia	NIB	1126	*Cotoneaster* sp.	2007
Slovenia	NIB	1130	Apple	2007
Slovenia	NIB	1184	Apple	2007
Slovenia	NIB	1200	Pear	2008
Slovenia	NIB	1208	Apple	2008
Slovenia	NIB	1211	Apple	2008
Slovenia	NIB	1214	Apple	2008
Slovenia	NIB	1216	*Cydonia* sp.	2008
Slovenia	NIB	1217	*Mespilus* sp.	2008
Slovenia	NIB	1223	Apple	2008
Slovenia	NIB	1224	Pear	2008
Slovenia	NIB	1259	Pear	2008
Slovenia	NIB	1260	Apple	2008
Slovenia	NIB	1263	Apple	2008
Slovenia	NIB	1334	Apple	2009
Slovenia	NIB	1335	Apple	2009
Slovenia	NIB	1339	Pear	2009
Spain	IVIA	IVIA 2887-21b	Pear	2004
Spain	IVIA	IVIA 2887-26	Pear	2004
Spain	IVIA	IVIA 2921-2	*Pyrus pyrifolia* (Nashi)	2004

Spain	IVIA	IVIA 2921-10	*Pyrus pyrifolia* (Nashi)	2004
Spain	IVIA	IVIA 3149	*Crataegus azarolus*	2006
Spain	IVIA	IVIA 3175	*Pyracantha* sp.	2006
Spain	IVIA	IVIA 3156 col. 12	Pear	2006
Spain	IVIA	IVIA 3156 col. 19	Pear	2006
Spain	IVIA	IVIA 3156 col. 20	Pear	2006
Spain	IVIA	IVIA 3156 col. 22	Pear	2006
Spain	IVIA	IVIA 2070-1	Pear	1999
Spain	IVIA	IVIA 2072-1	Pear	1999
Spain	IVIA	IVIA 2092	*Pyracantha* sp.	1999
Spain	IVIA	IVIA 2112-1	*Pyracantha* sp.	1999
Spain	IVIA	IVIA 2134-1	Pear	1999
Spain	IVIA	IVIA 2278-1	Pear	2000
Spain	IVIA	IVIA 2303-1	Apple	2000
Spain	IVIA	IVIA 2303-3	Pear	2000
Spain	IVIA	IVIA 2303-6	Pear	2000
Spain	IVIA	IVIA 2311-6	Pear	2000
Spain	IVIA	IVIA 2311-8	Pear	2000
Spain	IVIA	IVIA 2311-19	Pear	2000
Spain	IVIA	IVIA 2500	Apple	2001
Spain	IVIA	IVIA 2501	Pear	2001
Spain	IVIA	IVIA 1509-B	Apple	1995
Spain	IVIA	IVIA 1525-1	*Cotoneaster* sp.	1996
Spain	IVIA	IVIA 1525-6	*Cotoneaster* sp.	1996
Spain	IVIA	IVIA 1626	Apple	1996
Spain	IVIA	IVIA 1626-6	Apple	1996
Spain	IVIA	IVIA 1767-3	Apple	1997
Spain	IVIA	IVIA 1731-1	Pear	1997
Spain	IVIA	IVIA 1739-1	Apple	1997
Spain	IVIA	IVIA 1777-1	*Pyracantha* sp.	1997
Spain	IVIA	IVIA 1898-4	*Cotoneaster* sp.	1998
Spain	IVIA	IVIA 1899-21	*Cydonia oblonga*	1998
Spain	IVIA	IVIA1961-5	Pear	1998
Spain	IVIA	IVIA 1961-16	Pear	1998
Spain	IVIA	IVIA 1924-4	*Pyracantha* sp.	1998
Spain	IVIA	IVIA 1951-5	*Cotoneaster* sp.	1998
Spain	IVIA	IVIA 1951-8	*Pyracantha* sp.	1998
Spain	IVIA	IVIA 1951-6	*Sorbus* sp.	1998
Spain	IVIA	IVIA 1951-2	*Pyracantha* sp.	1998
Spain	UPN	UPN 529	*Pyracantha* sp.	1997
Spain	UPN	UPN 525	*Pyracantha* sp.	1997
Spain	UPN	UPN 526	Pear	1997
Spain	UPN	UPN 527	Apple	1997
Spain	UPN	UPN 528	Apple	1997
Spain	UPN	UPN 530	Pear	1997
Spain	UPN	UPN 500	Pear	1998
Spain	UPN	UPN 511	Apple	1998
Spain	UPN	UPN 537	*Cotoneaster* sp.	1998

Spain	UPN	UPN 540	Pear	1998
Spain	UPN	UPN 545	Apple	1998
Spain	UPN	UPN 546	*Cydonia oblonga*	1998
Spain	UPN	UPN 547	*Pyracantha* sp.	1998
Spain	UPN	UPN 564	*Cotoneaster* sp.	1998
Spain	UPN	UPN 582	*Pyracantha* sp.	1998
Spain	UPN	UPN 583	*Pyracantha* sp.	1998
Spain	UPN	UPN 584	*Pyracantha* sp.	1998
Spain	UPN	UPN 585	*Pyracantha* sp.	1998
Spain	UPN	UPN 586	*Pyracantha* sp.	1998
Spain	UPN	UPN 587	*Pyracantha* sp.	1998
Spain	UPN	UPN 588	*Pyracantha* sp.	1998
Spain	UPN	UPN 589	*Pyracantha* sp.	1998
Spain	UPN	UPN 590	*Pyracantha* sp.	1998
Spain	UPN	UPN 591	*Pyracantha* sp.	1998
Spain	UPN	UPN 592	*Pyracantha* sp.	1998
Spain	UPN	UPN 593	*Pyracantha* sp.	1998
Spain	UPN	UPN 594	*Pyracantha* sp.	1998
Spain	UPN	UPN 609	*Sorbus* sp.	1998
Spain	UPN	UPN 611	*Cotoneaster* sp.	1998
Spain	UPN	UPN 612	*Pyracantha* sp.	1998
Spain	UPN	UPN 613	na	Na
Spain	UPN	UPN 535	Apple	1998
Spain	UPN	UPN 536	Apple	1998
Spain	UPN	UPN 539	Pear	1998
Spain	UPN	UPN 541	*Pyracantha* sp.	1998
Spain	UPN	UPN 542	*Pyracantha* sp.	1998
Spain	UPN	UPN 543	*Pyracantha* sp.	1998
Spain	UPN	UPN 544	*Cotoneaster* sp.	1998
Spain	UPN	UPN 548	*Pyracantha* sp.	1998
Spain	UPN	UPN 549	*Pyracantha* sp.	1998
Spain	UPN	UPN 550	*Pyracantha* sp.	1998
Spain	UPN	UPN 551	*Pyracantha* sp.	1998
Spain	UPN	UPN 552	*Pyracantha* sp.	1998
Spain	UPN	UPN 553	*Pyracantha* sp.	1998
Spain	UPN	UPN 554	*Pyracantha* sp.	1998
Spain	UPN	UPN 555	*Pyracantha* sp.	1998
Spain	UPN	UPN 556	*Pyracantha* sp.	1998
Spain	UPN	UPN 557	*Pyracantha* sp.	1998
Spain	UPN	UPN 558	*Pyracantha* sp.	1998
Spain	UPN	UPN 559	Apple	1998
Spain	UPN	UPN 560	*Pyracantha* sp.	1998
Spain	UPN	UPN 561	*Pyracantha* sp.	1998
Spain	UPN	UPN 562	*Pyracantha* sp.	1998
Spain	UPN	UPN 563	*Pyracantha* sp.	1998
Spain	UPN	UPN 565	*Cotoneaster* sp.	1998
Spain	UPN	UPN 566	*Pyracantha* sp.	1998
Spain	UPN	UPN 567	*Pyracantha* sp.	1998

Spain	UPN	UPN 568	*Crataegus* sp.	1998
Spain	UPN	UPN 569	*Cotoneaster* sp.	1998
Spain	UPN	UPN 570	*Pyracantha* sp.	1998
Spain	UPN	UPN 571	Apple	1998
Spain	UPN	UPN 572	Apple	1998
Spain	UPN	UPN 573	*Pyracantha* sp.	1998
Spain	UPN	UPN 574	Apple	1998
Spain	UPN	UPN 575	Pear	1998
Spain	UPN	UPN 576	*Pyracantha* sp.	1998
Spain	UPN	UPN 577	*Pyracantha* sp.	1998
Spain	UPN	UPN 578	*Pyracantha* sp.	1998
Spain	UPN	UPN 579	*Pyracantha* sp.	1998
Spain	UPN	UPN 580	*Pyracantha* sp.	1998
Spain	UPN	UPN 581	*Pyracantha* sp.	1998
Spain	EPS	EPS 101	Pear	na
Spain	SV	SV 4576	Pear	na
Switzerland	ACW	57218	na	2007
Switzerland	ACW	57065	na	2007
Switzerland	ACW	57210	na	2007
Switzerland	ACW	57112	na	2007
Switzerland	ACW	59998	na	2007
Switzerland	ACW	60039	na	2007
Switzerland	ACW	59999	na	2007
Switzerland	ACW	59868	na	2007
Switzerland	ACW	59969	na	2007
Switzerland	ACW	59912	na	2007
Switzerland	ACW	59973	na	2007
Switzerland	ACW	59956	na	2007
Switzerland	ACW	58459	na	2007
Switzerland	ACW	56965	na	2007
Switzerland	ACW	56961	na	2007
Switzerland	ACW	56968	na	2007
Switzerland	ACW	57114	na	2007
Switzerland	ACW	57467	na	2007
Switzerland	ACW	56929	na	2007
Switzerland	ACW	56963	na	2007
Switzerland	ACW	60019	na	2007
Switzerland	ACW	57045	na	2007
Switzerland	ACW	59677	na	2007
Switzerland	ACW	59959	na	2007
Switzerland	ACW	56934	na	2007
Switzerland	ACW	57143	na	2007
Switzerland	ACW	57113	na	2007
Switzerland	ACW	59889	na	2007
Switzerland	ACW	59888	na	2007
Switzerland	ACW	59557	na	2007
Switzerland	ACW	56928	na	2007
Switzerland	ACW	57488	na	2007

Switzerland	ACW	57111	na	2007
Switzerland	ACW	57078	na	2007
Switzerland	ACW	56932	na	2007
Switzerland	ACW	60021	na	2007
Switzerland	ACW	59717	na	2007
Switzerland	ACW	57575	na	2007
Switzerland	ACW	59987	na	2007
Switzerland	ACW	56839	na	2007
Switzerland	ACW	56508	na	2007
Switzerland	ACW	56867	na	2007
Switzerland	ACW	56823	na	2007
Switzerland	ACW	56310	na	2007
Switzerland	ACW	56216	na	2007
Switzerland	ACW	56657	na	2007
Switzerland	ACW	56363	na	2007
Switzerland	ACW	57117	na	2007
Switzerland	ACW	57560	na	2007
Switzerland	ACW	59690	na	2007
Switzerland	ACW	56933	na	2007
Switzerland	ACW	56098	na	2007
Switzerland	ACW	57546	na	2007
Switzerland	ACW	56847	na	2007
Switzerland	ACW	56283	na	2007
Switzerland	ACW	56486	na	2007
Switzerland	ACW	56842	na	2007
Switzerland	ACW	56511	na	2007
Switzerland	ACW	56959	na	2007
Switzerland	ACW	56948	na	2007
Switzerland	ACW	56930	na	2007
Switzerland	ACW	57132	na	2007
Switzerland	ACW	59517	na	2007
Switzerland	ACW	56605	na	2007
Switzerland	ACW	56848	na	2007
Switzerland	ACW	56121	na	2007
Switzerland	ACW	56120	na	2007
Switzerland	ACW	56387	na	2007
Switzerland	ACW	59910	na	2007
Switzerland	ACW	57506	na	2007
Switzerland	ACW	57561	na	2007
Switzerland	ACW	56902	na	2007
Switzerland	ACW	56933	Pear	2007
Switzerland	ACW	56232	na	2007
Switzerland	ACW	56482	na	2007
Switzerland	ACW	56601	na	2007
Switzerland	ACW	55792	na	2007
Switzerland	ACW	56199	na	2007
Switzerland	ACW	57116	na	2007
Switzerland	ACW	59718	na	2007

Switzerland	ACW	57530	na	2007
Switzerland	ACW	56934	na	2007
Switzerland	ACW	57479	na	2007
Switzerland	ACW	56437	na	2007
Switzerland	ACW	56499	na	2007
Switzerland	ACW	56298	Pear	2007
Switzerland	ACW	56200	na	2007
Switzerland	ACW	56785	na	2007
Switzerland	ACW	56762	na	2007
Switzerland	ACW	56099	na	2007
Switzerland	ACW	56177	na	2007
Switzerland	ACW	56187	na	2007
Switzerland	ACW	38158	na	2007
Switzerland	ACW	39011	na	2007
Switzerland	ACW	45021	na	2007
Switzerland	ACW	RAC 3077	na	2007
Switzerland	ACW	38291	na	2007
Switzerland	ACW	39010	na	2007
Switzerland	ACW	37803+	na	2007
Switzerland	ACW	37602	na	2007
Switzerland	ACW	43577	na	2007
Switzerland	ACW	43579	na	2007
Switzerland	ACW	43802	na	2007
Switzerland	ACW	35734	na	2007
Switzerland	ACW	43590	na	2007
Switzerland	ACW	35696	na	2007
Switzerland	ACW	35697	na	2007
Switzerland	ACW	35698	na	2007
Switzerland	ACW	35731	na	2007
Switzerland	ACW	35683	na	2007
Switzerland	ACW	35684	na	2007
Switzerland	ACW	35686	na	2007
Switzerland	ACW	35689	na	2007
Switzerland	ACW	39005	na	2007
Switzerland	ACW	38900	na	2007
Switzerland	ACW	37915+	na	2007
Switzerland	ACW	37783+	na	2007
Switzerland	ACW	37686	na	2007
Switzerland	ACW	39002	na	2007
Switzerland	ACW	38249	na	2007
Switzerland	ACW	38878	na	2007
Switzerland	ACW	44567	na	2007
Switzerland	ACW	45015	na	2007
Switzerland	ACW	44432	na	2007
Switzerland	ACW	44736	na	2007
Switzerland	ACW	45534	na	2007
Switzerland	ACW	45009	na	2007
Switzerland	ACW	45034	na	2007

Switzerland	ACW	44193	na	2007
Switzerland	ACW	45069	na	2007
Switzerland	ACW	45962	na	2007
Switzerland	ACW	37607	na	2007
Switzerland	ACW	37812	na	2007
Switzerland	ACW	37825	na	2007
Switzerland	ACW	37920+	na	2007
Switzerland	ACW	RAC 2875	na	2007
Switzerland	ACW	RAC 99/85	na	2007
Switzerland	ACW	RAC 3080	na	2007
Switzerland	ACW	RAC 2235	na	2007
Switzerland	ACW	RAC 3072	na	2007
Switzerland	ACW	RAC 3074 B	na	2007
Switzerland	ACW	36915	na	2007
Switzerland	ACW	36916	na	2007
Switzerland	ACW	36670	na	2007
Switzerland	ACW	37600+	na	2007
Switzerland	ACW	37905+	na	2007
Switzerland	ACW	38019	na	2007
Switzerland	ACW	38925	na	2007
Switzerland	ACW	42919	na	2007
Switzerland	ACW	37706	na	2007
Switzerland	ACW	36076	na	2007
Switzerland	ACW	36694	na	2007
Switzerland	ACW	36670	na	2007
Switzerland	ACW	RAC 2414	na	2007
Switzerland	ACW	42918	na	2007
Switzerland	ACW	39012	na	2007
Switzerland	ACW	39018	na	2007
Switzerland	ACW	37811	na	2007
Switzerland	ACW	37623	na	2007
Switzerland	ACW	36097	na	2007
Switzerland	ACW	36064	na	2007
Switzerland	ACW	35978	na	2007
Switzerland	ACW	42306	na	2007
Switzerland	ACW	42915	na	2007
Switzerland	ACW	45008	na	2007
Switzerland	ACW	42906	na	2007
Switzerland	ACW	42951	na	2007
Switzerland	ACW	36056	na	2007
Switzerland	ACW	37830+	na	2007
Switzerland	ACW	37838	na	2007
Switzerland	ACW	39000	na	2007
Switzerland	ACW	39015	na	2007
Switzerland	ACW	42907	na	2007
Switzerland	ACW	42913	na	2007
Switzerland	ACW	37884	na	2007
Switzerland	ACW	39004	na	2007

Switzerland	ACW	35506	na	2007
Switzerland	ACW	28357	na	2007
Switzerland	ACW	32953	na	2007
Switzerland	ACW	35065	na	2007
Switzerland	ACW	na	na	2007
Switzerland	ACW	na	na	2007
Switzerland	ACW	35301	na	2007
Switzerland	ACW	35294	na	2007
Switzerland	ACW	35291	na	2007
Switzerland	ACW	35295	na	2007
Switzerland	ACW	35408	na	2007
Switzerland	ACW	35367	na	2007
Switzerland	ACW	35368	na	2007
Switzerland	ACW	35391	na	2007
Switzerland	ACW	35409	na	2007
Switzerland	ACW	35300	na	2007
Switzerland	ACW	35303	na	2007
Switzerland	ACW	35302	na	2007
Switzerland	ACW	35299	na	2007
Switzerland	ACW	36629	na	2007
Switzerland	ACW	35396	na	2007
Switzerland	ACW	35402	na	2007
Switzerland	ACW	35389	na	2007
Switzerland	ACW	35484	na	2007
Switzerland	ACW	35522	na	2007
Switzerland	ACW	35524	na	2007
Switzerland	ACW	35529	na	2007
Switzerland	ACW	35366	na	2007
Switzerland	ACW	35357	na	2007
Switzerland	ACW	35356	na	2007
Switzerland	ACW	35418	na	2007
Switzerland	ACW	35393	na	2007
Switzerland	ACW	35377	na	2007
Switzerland	ACW	35407	na	2007
Switzerland	ACW	35297	na	2007
Switzerland	ACW	35462	na	2007
Switzerland	ACW	35289	na	2007
Switzerland	ACW	35306	na	2007
Switzerland	ACW	35307	na	2007
Switzerland	ACW	35298	na	2007
Switzerland	ACW	35312	na	2007
Switzerland	ACW	35299	na	2007
Switzerland	ACW	35388	na	2007
Switzerland	ACW	35385	na	2007
Switzerland	ACW	35476	na	2007
Switzerland	ACW	32920	na	2007
Switzerland	ACW	32953	na	2007
Switzerland	ACW	32945	na	2007

Switzerland	ACW	34978	na	2007
Switzerland	ACW	34998	na	2007
Switzerland	ACW	34945	na	2007
Switzerland	ACW	32953	na	2007
Switzerland	ACW	34962	na	2007
Switzerland	ACW	35145	na	2007
Switzerland	ACW	35091	na	2007
Switzerland	ACW	35063	na	2007
Switzerland	ACW	35065	na	2007
Switzerland	ACW	35075	na	2007
Switzerland	ACW	35270	na	2007
Switzerland	ACW	35266	na	2007
Switzerland	ACW	35250	na	2007
Switzerland	ACW	35264	na	2007
Switzerland	ACW	35263	na	2007
Switzerland	ACW	35060	na	2007
Switzerland	ACW	35260	na	2007
Switzerland	ACW	35205	na	2007
Switzerland	ACW	35281	na	2007
Switzerland	ACW	39203	na	2007
The Netherlands	CFBP	CFBP 3020	Pear	1981
The Netherlands	NCPPB	NCPPB 3159	Apple	1980
UK	CFBP	CFBP 3041	Pear	na
UK	CFBP	CFBP 3042	Apple	na
UK	BBA	KG P-1573	*Cotoneaster* sp.	na
UK	BBA	KG 98 3042	Apple	na
UK	NCPPB	NCPPB 595	Pear	1958
UK	NCPPB	NCPPB 683	Pear	1959

na: not available.

ACW: Agroscope-Changins Wädenswil, Switzerland.

AGES: Austrian Agency for Health and Food Safety. Wien, Austria.

ARI: Agricultural Research Institute SERBIA - Pesticide and Environmental Research Centre, Belgrade-Zemun. Serbia.

BBA: Federal Biological Institute for Agriculture and Forestry. Dossenheim, Germany.

BPIC: Benaki Phytopathological Institute Collection, Athens, Greece.

CFBP: Collection Française de Bactéries Phytopathogènes, INRA, Angers, France.

CRA-W : Centre Wallon de Recherches agronomiques. Gembloux, Belgium.

EPS: Escola Politècnica Superior-Universitat de Girona, Spain.

INRA : Institut National de la Recherche Agronomique, UMR de Pathologie Végétale , Beaucouzé,France

IVIA: Instituto Valenciano de Investigaciones Agrarias collection, Moncada, Spain

LMG: Collection of the Laboratory of Microbiology. Rijksuniversiteit, Gent, Belgium

LNPV: Laboratoire National de la Protection des Végétaux, Beaucouzé, France.

NCPPB: National Collection of Plant Pathogenic Bacteria, York, UK.

NIB: National Institute of Biology, Ljubljana, Slovenia.

OMP: Osservatorio per le Malattie delle Piante, Bologna, Italy.

SL: State Laboratory, Dublin, Ireland.

SV: Servei de Protecció dels Vegetals-Departament d'Agricultura, Ramaderia i Pesca de la Generalitat de Catalunya, Barcelona, Spain.

UPN : Universidad Publica de Navarra. Pamplona, Spain.
